# Water doping sodium battery electrolyte controls nanostructure, interactions, and electrochemical properties

**DOI:** 10.1126/sciadv.aee3415

**Published:** 2026-05-29

**Authors:** Xuhui Zhang, Qianlu Zheng, Hua Li, Zachary A. H. Goodwin, Alexis G. Hoane, Alexander Deptula, Owen M. Johnson, Juhyun Song, Daniel M. Markiewitz, Martin Z. Bazant, Cecilia Leal, Filippo Mangolini, Andrew A. Gewirth, Rob Atkin, Mark W. Rutland, Rosa M. Espinosa-Marzal

**Affiliations:** ^1^ Department of Civil and Environmental Engineering, University of Illinois at Urbana-Champaign, Urbana, IL 61801, USA.; ^2^ School of Molecular Sciences, The University of Western Australia, Perth, Western Australia 6009, Australia.; ^3^ Centre for Microscopy, Characterisation and Analysis, The University of Western Australia, Perth, Western Australia 6009, Australia.; ^4^ John A. Paulson School of Engineering and Applied Sciences, Harvard University, Cambridge, MA 02138, USA.; ^5^ Department of Materials, University of Oxford, Parks Road, Oxford OX1 3PH, UK.; ^6^ Department of Chemistry, University of Illinois at Urbana-Champaign, Urbana, IL 61801, USA.; ^7^ Department of Materials Science and Engineering, University of Illinois at Urbana-Champaign, Urbana, IL 61801, USA.; ^8^ Walker Department of Mechanical Engineering, The University of Texas at Austin, Austin, TX 78712, USA.; ^9^ Department of Energy Engineering, Korea Institute of Energy Technology, Naju, Jeonnam 58330, Republic of Korea.; ^10^ Department of Chemical Engineering, Massachusetts Institute of Technology, Cambridge, MA 02139, USA.; ^11^ Department of Mathematics, Massachusetts Institute of Technology, Cambridge, MA 02139, USA.; ^12^ Department of Chemistry, KTH Royal Institute of Technology, Stockholm SE-100 44, Sweden.; ^13^ School of Chemistry, University of New South Wales, Sydney 2052, Australia.; ^14^ Laboratoire de Tribologie et Dynamique des Systèmes, École Centrale de Lyon, Lyon 69130, France.; ^15^ Bioeconomy and Health, Materials and Surface Design, RISE (Research Institutes of Sweden), Stockholm 114 28, Sweden.

## Abstract

Salt-in-ionic liquids (SiILs) are promising electrolytes for batteries. This study reveals how water affects the nanostructure, surface forces, and electrochemical properties of sodium-SiILs with bis(trifluoromethanesulfonyl)imide ([TFSI]^−^) using experiments and molecular dynamics simulations. Dry sodium-SiILs exhibit long-range repulsive forces that deviate from classical electrostatics and are influenced by surface-induced aggregation of nanoscale ionic clusters. Addition of water reduces cluster size and order, yielding force profiles more similar to neat ILs. Atomic force microscopy shows water-induced cluster reorganization near negatively charged surfaces. Water-in-SiILs exhibit increased capacitance and a shift from camel- to bell-shaped profiles, indicating a fundamental change in the double layer, while enhancing conductivity and maintaining a wide electrochemical stability window. These findings underscore the sensitivity of the SiIL nanostructure to hydration from bulk to interface and its critical role in electrochemical properties. Advances in the understanding of the interplay between the nanostructure and screening are essential for the rational design of the solid electrolyte interphase, a crucial component dictating battery performance and safety.

## Teaser

Water doping in sodium battery electrolyte controls nanostructure, interactions, and electrochemical properties.

## INTRODUCTION

The electrolyte plays a central role in battery operation, strongly influencing energy density and cycle life (*1*, *2*). Electrolytes with a super-high concentration have emerged as an effective strategy to enable high-energy-density storage and to modulate the formation of the solid-electrolyte interphase (SEI). The SEI, a passivation layer that forms on the electrode surface through electrolyte decomposition, plays a critical role in battery performance and safety by regulating ion transport and preventing further electrolyte decomposition. In this context, ionic liquids (ILs) are a promising class of solvents because of their unique combination of properties, such as chemical versatility, extremely low vapor pressures, and generally low flammability (*3*). Accordingly, doping ILs with alkali-metal salts yields a compelling electrolyte platform for battery research, primarily because of enhanced safety relative to conventional organic solvents and the possibility of tuning SEI formation and its properties in metal-ion batteries, including systems beyond lithium (*4*–*7*).

In these so-called salt-in-ILs (SiILs), experiments and molecular dynamics (MD) simulations have found negative transference numbers at low mole fractions of alkali metal salts (*8*–*10*). While this was a puzzling observation initially, such behavior can be explained by the formation of negatively charged clusters composed of alkali metal cations and anions (*11*). Notably, MD simulations have also shown the formation of a percolating ionic network at higher mole fractions of alkali metal salts, which leads to net positive transference numbers (*11*). There is evidence from experiments and simulations of the existence of aggregation in other concentrated electrolytes (*12*–*14*), although some studies have questioned whether ionic aggregates are responsible for negative transference numbers (*15*, *16*). Regardless of this debate, the existence and importance of ionic aggregation in SiILs are well documented (*17*).

Recently, we investigated a dry Na-based SiIL with 1-ethyl-3-methylimidazolium bis(trifluoromethanesulfonyl)imide ([EMIM][TFSI]) as the IL (*18*). Surface forces apparatus (SFA) measurements showed long-range repulsive forces between mica surfaces, with an onset as far as ∼100 nm. Long-range surface forces in highly concentrated electrolytes were first reported for ILs (*19*, *20*) and proposed to be of electrostatic origin (*19*). The large decay length (up to ∼10 nm) was explained by the renormalization of the charge carrier concentration (*21*). This yields a screening length much larger than the Debye length, surpassing the expected underscreening of highly concentrated electrolytes according to theory (*22*) and leading to the concept of anomalous underscreening (*23*). The surface forces in this dry SiIL show characteristics that cannot be explained by this model: the “forced separation” of the mica surfaces upon the first approach of the surfaces and the highly reproducible difference between the first and subsequent approaches. This leads to two decay lengths, $d_{1}$ (first approach) and $d_{2}$ (subsequent approaches) that increase with sodium bis(trifluoromethanesulfonyl)imide (NaTFSI) content. We found evidence of surface and confinement-induced structural changes yielding nanostructures of larger size than in the bulk, thereby justifying the increase from $d_{1}$ to $d_{2}$. The influence of equilibration time and approach rate pointed at slow dynamics in the SiILs and led to the conclusion that the long-ranged surface forces in SiILs are not purely electrostatic in origin. Rather, they are influenced by the compression of high-aspect-ratio nanostructures that gradually form at the interfaces with long relaxation/equilibration times, and therefore, they cannot be described by simple charge-carrier renormalization. A recent study has revealed the contribution of dynamics (hydrodynamic) effects to the long-range forces in neat ILs (*24*).

Although there are limited detailed studies of battery performance using water-in-SiILs as electrolytes, a few studies demonstrate the substantial impact of the water content on the SEI composition and properties (*25*, *26*). The presence of water promoted the formation of a more uniform SEI on Na-metal anode surfaces with a distinct chemical composition and microstructure compared to the dry electrolyte (*26*). Although the average cycling efficiency increased only modestly, from 98% up to ∼99%, the SEI that formed in the wet electrolyte was more stable, compact, and uniform, which was associated with a more stable symmetric cell voltage profile and reduced cell polarization relative to the dry electrolyte. In contrast, the higher fraction of microporous deposits and the absence of a compact layer in the SEI formed in the dry SiIL correlated with increased cell polarization potentials and the occurrence of dendritic growth, emphasizing the relevance of the interfacial water content. This work thus investigates how substituting a fraction of the IL with water in a Na-SiIL electrolyte enables tunable bulk and interfacial structuring, long-range interactions, and electrochemical properties, providing insight into a pathway for enhanced battery properties.

## RESULTS

NaTFSI (97%, RoCo) and the IL [EMIM][TFSI] (97%, Sigma-Aldrich) were dried at 50°C under vacuum for more than 48 hours. Although other ILs—e.g., those based on pyrrolidinium cations and bis(fluorosulfonyl)imide ([FSI]^−^) anions—have shown greater promise for battery applications (*4*, *27*), at least in the dry state, [EMIM][TFSI] was selected for this study because it enables a direct comparison between experimental results and MD simulations (*10*, *18*, *28*–*30*). In addition, the bis(trifluoromethanesulfonyl)imide ([TFSI]^−^) anion exhibits higher hydrolytic stability than [FSI]^−^ (*31*, *32*). The dry SiIL samples were prepared under a nitrogen gas (N_2_) atmosphere with a mole fraction ($x_{s}$) of 0.10 of NaTFSI and stored at room temperature with relative humidity (RH) values of 11 and 33% RH; this led to water uptake and equilibrium mole fractions ($x_{w}$) of 0.110 ± 0.002 and 0.289 ± 0.002, respectively (abbreviated as 0.1 and 0.3), greater than the trace amounts of water in the dry SiIL ($x_{w}$ = 0.003 ± 0.001) and in the neat IL (0.007 ± 0.003). Although the total mole fraction of salt in the water-in-SiILs decreases to 0.09 and 0.07, respectively, we nonetheless label the salt content as $x_{s}$ ∼ 0.1 for simplicity.

### SiIL structure from MD simulations and wide-angle x-ray scattering

**Fig. 1.**
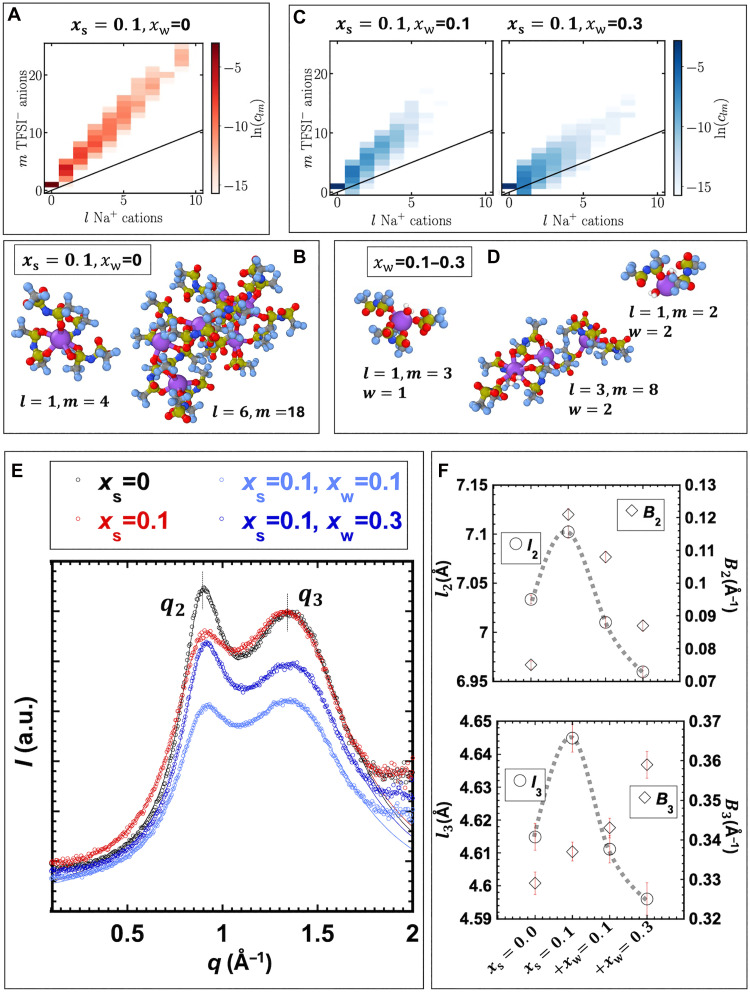
MD simulations and WAXS of the IL, SiIL, and water-in-SiILs. (**A** to **D**) MD simulation results. Additional results are shown in figs. S1 to S5. Concentrations of each cluster containing l Na^+^ cations and m [TFSI]^−^ anions in the (A) dry SiIL and (C) water-in-SiILs. (B) Most common aggregate in the dry SiIL, one Na^+^ with four [TFSI]^−^, and an example of a larger aggregate. (D) Examples of aggregates containing water. (**E**) Integrated intensity from WAXS two-dimensional scans. The thin black lines show the fits of Lorentzian functions. a.u., arbitrary units. (**F**) The fits of Lorentzian functions to the integrated WAXS intensities provide the *d*-spacing (l) and the half-width at half-maximum values (B) for reflections at $q_{2}$ and $q_{3}$. The B values were corrected with the instrument divergence. The fitting parameters can be found in table S1.

To gain insight into how the structure of the SiIL depends on the water content, we performed MD simulations of these compositions. The details of these simulations can be found in the Supplementary Materials. As previously reported in (*18*), the dry SiIL at $x_{s}=0.1$ contains a substantial number of ionic aggregates. To determine the associations in aggregates, we used a real-space cutoff criterion between Na^+^ and O atoms in [TFSI]^−^, while the associations between [EMIM]^+^ and [TFSI]^−^ were not considered, as they are weaker (*3*, *9*). Figure 1A shows the concentration of clusters containing l Na^+^ cations and m [TFSI]^−^ anions. For the dry SiIL, the most prevalent cluster contains one Na^+^ cation with four [TFSI]^−^ anions, which has a length scale of ∼12 Å, and a smaller number of clusters with one Na^+^ cation are bound to three or five [TFSI]^−^ anions (see also fig. S1). The average Na-TFSI coordination number is ∼3.9. There is a smaller population of larger ionic clusters, containing tens of ions. Figure 1B illustrates one such large cluster, which resembles a nanoparticle; other representative clusters are shown in fig. S2. The cluster size analysis in Fig. 1A reveals intermediate cluster sizes containing two Na^+^ cations and six to seven [TFSI]^−^ anions. The most frequent aggregate in the dry SiIL contains five ions, with a large number of clusters of the order of 7 to 12 ions (up to a size of ∼17 Å). While much less frequent, there is a continuum of cluster sizes up to 28 ions.

Upon the addition of water ($x_{w}$ of 0.1 and 0.3), MD simulations show that the distribution of ionic clusters becomes narrower (Fig. 1C). Representative clusters are also shown in Fig. 1D and fig. S2. In addition, the average number of ions in the clusters decreases overall and more so with the increase in water content (Fig. 1C). This is expected, as water binds strongly to (solvates) Na^+^ and weakens Na-TFSI interactions (*11*, *12*, *33*); again, we use a real-space cutoff criterion to determine whether a water molecule solvates Na^+^, as outlined in the Supplementary Materials. At the water content of $x_{w}=0.1$, the probability of three [TFSI]^−^ anions per Na^+^ approaches that of four [TFSI]^−^ anions (see fig. S1). There is now also a nonzero probability of Na-TFSI ion pairs, which are defined as ionic associations composed of one Na^+^ and one [TFSI]^−^. Hence, the average Na-TFSI coordination number decreases to 3.4 in water-in-SiILs with $x_{w}$ of 0.1. Figure S3 shows that there are still many Na^+^ cations that do not bind to water, but if they do bind, it is most frequently to one single water molecule. The size of the most frequent cluster (l = 1, m = 3, and w = 1) is ∼13 Å.

Increasing the water content to $x_{w}=0.3$ leads to a further decrease in the Na-TFSI coordination number to 2.04. The most frequent coordination environment of a Na^+^ cation is two [TFSI]^−^ anions and two water molecules, consistent with the increase in water content (figs. S1 and S3). The size of the most frequent cluster (l = 1, m = 2, and w = 2) is ∼9 Å. Only a very small fraction of Na^+^ cations does not coordinate with water at $x_{w}=0.3$. Simulations also show that about 60 and 65% of the water molecules are not bound to Na^+^ cations in the water-in-SiILs with $x_{w}$ = 0.1 and 0.3, respectively. These water molecules could interact with both [TFSI]^−^ and [EMIM]^+^ via hydrogen bonding, although this interaction was not investigated by MD simulations.

We also computed the cluster bond density (CBD), i.e., the number of associations in a cluster over the number of ions in that cluster (fig. S4). If this ratio is larger than the Cayley tree limit of disordered aggregates, there must be closed paths of associations in the cluster, so-called loops. The CBD is thus a measure of how ordered the aggregates are, with larger values corresponding to more looped, i.e., more ordered, aggregates; representative loops are shown in fig. S5. Overall, the CBD values are slightly above 1, which indicates the presence of loops in the ionic aggregates. With the addition of water, the CBD values decrease, especially for larger aggregates. Moreover, with increasing water content $x_{w}$ to 0.3, the CBD is almost only on the Cayley tree limit of disordered aggregates. This means that instead of resembling nanoparticles, the clusters have a more branched structure. In Fig. 1D, we show an example of such aggregates, where several water molecules are shown to bind to the branched clusters. Overall, the addition of water leads, on average, to fewer ions in clusters, but these aggregates are more branched than in the case of the dry SiIL.

Figure 1E compares the integrated wide-angle x-ray scattering (WAXS) intensity for [EMIM][TFSI] with that of the dry SiIL and the water-in-SiILs. The superposition of two Lorentzian functions was used to fit the scattering intensity: $\sum_{i=1}^{N}\frac{A_{i}}{1+{\left(\frac{q-q_{i}}{B_{i}}\right)}^{2}}$, where $A_{i}$ is the intensity factor, $q_{i}$ is the peak position, and $B_{i}$ is the half-width at half-maximum, which was corrected with instrumental broadening. The fitting parameters and their errors are shown in table S1.

Two reflections are observed for the neat IL, one at $q_{2}$ ∼ 0.89 Å^−1^ and the other at $q_{3}$ ∼ 1.36 Å^−1^. In pure ILs (*34*), the peak at the lowest *q* gives the length scale of the nanoscopic heterogeneities arising from alternating charged and uncharged domains ($q_{1}$) typically associated with solvophobic self-assembly; the charge alternation peak at mid-*q* corresponds to correlations between charged groups in the polar domain ($q_{2}$), and the adjacency peak at higher q results from the interactions between neighboring ions ($q_{3}$). The absence of peak $q_{1}$ for [EMIM][TFSI] is in agreement with previous work (*35*) and is attributed to the short alkyl chain in the cation, which weakens solvophobic interactions. For both the dry SiIL and water-in-SiILs, the reflection at $q_{1}$ remains undetectable, indicating the absence of nanoscopic heterogeneities. It is possible that the wide distribution of cluster sizes, as verified in MD simulations, prevents these heterogeneities from being detected. Moreover, trace amounts of water in the nominally dry SiIL may introduce additional disorder.

The correlation lengths corresponding to the reflections at $q_{2}$ and $q_{3}$ ($l_{i}=2\pi /q_{i}$) are influenced by the addition of salt and water, reflecting changes in interionic distances (see Fig. 1F). Specifically, $l_{2}$ increases from 7.033 to 7.102 Å when salt is added to the dry IL, and it decreases to 7.010 at $x_{w}$ = 0.1 and to 6.959 Å at $x_{w}$ = 0.3. A similar expansion upon addition of salt and contraction with an increase in water content are observed for $l_{3}$ (4.615, 4.645, 4.611, and 4.596 Å, respectively). The fits also show that the half-width at half-maximum value of $q_{2}$ and $q_{3}$ changes with composition. The addition of salt ($x_{s}$ = 0.1) leads to more disorder in both alternating and adjacency peaks, i.e., $q_{2}$ and $q_{3}$ become broader relative to [EMIM][TFSI]. However, adding water decreases the broadening at $q_{2}$, indicating that the correlation between same-charge ions is better organized than in the dry SiIL yet less than in the pure IL. In contrast, the broadening of $q_{3}$ increases gradually with the addition of water. Together, the results from WAXS indicate that the addition of water reduces interionic distances relative to the dry SiIL (and the neat IL), which coincides with the reduced ion clustering and more branched (disordered) structures in the humid electrolyte inferred from MD simulations.

### Surface forces under confinement

**Fig. 2.**
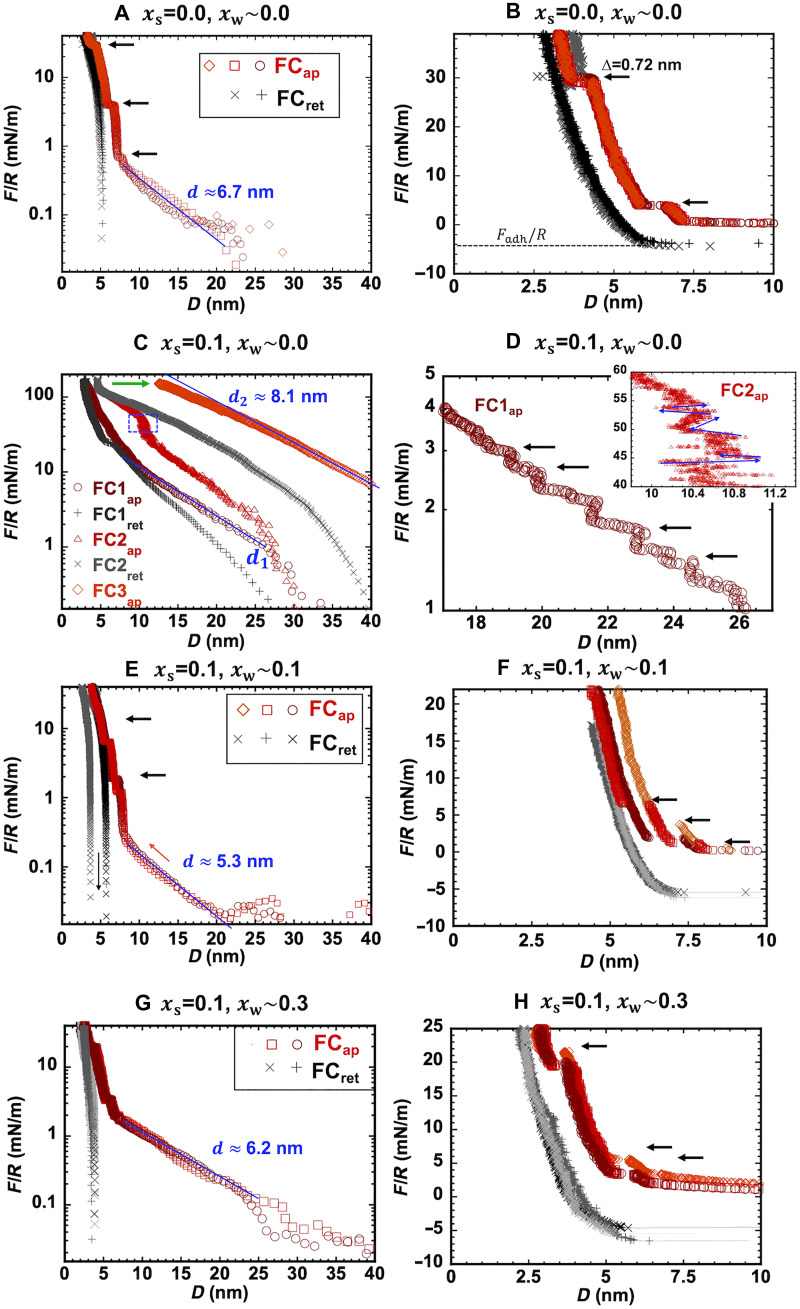
Representative surface forces between mica surfaces in neat IL, SiIL with $x_{s}$ = 0.1, and water-in-SiILs with $x_{w}$ = 0.1 and $x_{w}$ = 0.3, all at 25°C. (**A** and **B**) Force versus surface separation in neat [EMIM][TFSI] ($x_{s}$ = 0) equilibrated at RH smaller than 3%; the empty red symbols represent the force upon approach of the surfaces, and the black crosses represent the force upon retraction. Force-distance curves in (**C** and **D**) dry SiIL with a salt (NaTFSI) mole fraction ($x_{s}$) of 0.10 (<3% RH) and water-in-SiILs with (**E** and **F**) $x_{w}$ ∼ 0.1 (11% RH) and (**G** and **H**) $x_{w}$ ∼ 0.3 (33% RH). The blue lines show exponential fits with decay length d. Each force-distance curve displays the force normalized by the radius of the surface, F/R, versus the surface separation, D. Note the different *y* axis of (B) compared to those of (A), (E), and (G).

Force measurements between mica surfaces were carried out using an SFA (see details in the Supplementary Materials text). For the neat IL, the results in Fig. 2A agree well with the reported results (*36*). At surface separations (D) larger than ∼8 nm, the surface force decreases quasi-exponentially with D having a decay length (d) of ∼6.9 ± 1 nm. Figure 2A shows good agreement between three consecutive force-distance curves. At surface separations smaller than ∼8 nm, discrete steps were observed in all force-separation curves (see Fig. 2B). These steps reflect the layered arrangement of ions near the mica surfaces, giving rise to an oscillatory structural (solvation) force. Each step thus represents the squeeze-out of a layer of ions, with a thickness of ∆ ∼ 7.2 Å, which is close to the diameter of an ion pair (estimated from the cube root of the ion-pair volume, 7.5 Å), and therefore, it defines the characteristic length scale of a charge-neutral layer. A pull-off force of ∼4.1 ± 0.4 mN/m is measured upon retraction of the surfaces.

Figure 2C shows the results for the dry SiIL. A characteristic of this electrolyte is that there is a difference between consecutive force-distance curves. Thus, the first approach and retraction curves are labeled as “FC1_ap_” and “FC1_ret_,” respectively. The subsequent force-distance curves are labeled as “FC2_ap_” and “FC2_ret_” for the approach and retraction, respectively. All surface forces are repulsive, and hence, there is no pull-off force to report for this SiIL. Over intermediate surface separations, FC1 increases quasi-exponentially with decreasing D, with a decay length of $d_{1}$ ∼ 5.3 ± 1.6 nm; the blue line shows $d_{1}$ ∼ 6.7 nm. Note that the extracted decay length depends strongly on the fitting range, as the force profile is not well described by a single exponential; therefore, the absolute values should be interpreted with caution. A careful inspection of the force-distance curves reveals steps with a size of ∆ = 11.4 ± 4.7 Å at D > 10 nm, which is reminiscent of the cluster size inferred from MD simulations. Upon retraction, the force profile FC1_ret_ remains repulsive but displays hysteresis, suggesting that dynamic effects influence the measured behavior. The subsequent force-distance curve, FC2_ap_, deviates from FC1_ap_. At a surface separation D of ∼10.5 nm in FC2_ap_, the force increases steeply from F/R ∼ 30 to 55 mN/m, while D remains approximately constant (see this transition in the blue box in Fig. 2C). Thereafter, the force increases as the surface separation decreases. A clockwise hysteresis is observed upon retraction, indicating a change of the interfacial structure. The subsequent force-distance curves, exemplified by FC3_ap_, are of longer range, with an exponential decay over intermediate surface separations, $d_{2}$ ∼8.2 ± 1.5 nm, and do not exhibit steps. In contrast to the pure IL, the force profiles FC1 to FC3 show variability, with changes in both the decay length and force onset.

Many of the features described above are characteristic of this dry SiIL with $x_{s}$ from 0.008 to ∼0.2 NaTFSI (saturation), as reported in (*18*). That previous work reported surface force measurements carried out at a faster velocity of approach and separation (0.3 nm/s). Instead of the upward kink described above (blue box in Fig. 2C), a faster approach led to a prominent outward kink in FC1_ap_, i.e., a forced separation against the driving direction over a distance of several nanometers. This earlier study also showed that the forced separation is either more subtle or absent (more often) at longer equilibration times (12 versus 24 hours) and slower approach velocities (like those used in this work). A closer look of the upward kink shows a series of outward steps, reminiscent of the forced separation in that work (see inset in Fig. 2D).

Despite the more or less prominent forced separation—depending on the equilibration time and the approach velocity—the force-distance curves of this dry SiIL are consistently of long range, with profiles that deviate from a strictly exponentially decaying force and with a consistent increase from $d_{1}$ to $d_{2}$. In our previous study (*18*), we were able to rule out artifacts from elastohydrodynamic distortion of the surface and pinning forces. We concluded that, initially, the approach of the surfaces forces the ionic clusters in the bulk electrolyte to arrange in layers, so they are slowly squeezed out as the surfaces approach, causing the nanometer large subtle steps. The enrichment of sodium near the negatively charged surfaces leads to surface- and confinement-promoted aggregation, which causes clusters with a larger characteristic length to form. This transition explains the increase in both the decay length to $d_{2}$ and onset of the force, and results in an outward/upward kink in the force profile. After the transition to *d*_2_, the resistance against the compression of the larger aggregates gives rise to a longer-range repulsive force.

The dependence on the approach rate and the differences between consecutive force-distance profiles indicate a substantial contribution of dynamics to the long-range surface force, pointing to the slow squeeze-out of the electrolyte and the sluggish kinetics of the surface/confinement-enhanced aggregation as it transitions gradually toward equilibrium. This is also consistent with the very long relaxation times inferred from simulations for a SiIL of different compositions (*9*, *37*). Furthermore, the comparison to results with other pairs of mica surfaces reflects the variability of the behavior, as (i) the decay length of the force profiles varies and (ii) the structural transition from $d_{1}$ to a larger decay length, $d_{2}$, is not always accompanied by a distinct kink in the force profile (see fig. S6). This variation presumably originates from the kinetics of the structural transition combined with the dynamic nature of our SFA measurements.

The characteristic behavior of water-in-SiILs containing a water mole fraction of $x_{w}$ ∼ 0.1 is displayed in Fig. 2E. The force-distance curves are markedly different from the results of the dry SiIL. First, they reveal multiple minima upon retraction as a result of the quantized adhesion between the two mica surfaces (*38*); the pull-off force at one of these minima is displayed in Fig. 2F (see also fig. S7).

Second, the approach curves display a quasi-exponential decay, and subsequent force-distance curves (FC1 to FC3) are essentially superimposable, in marked contrast to the case of dry SiIL. The decay length of 6.12 ± 1.23 nm is slightly smaller than that of the pure IL. The variability across experiments with different pairs of mica surfaces is reflected in the standard deviation. The force profiles exhibit multiple steps at D < 10 nm that indicate a layered structure in proximity of the mica surface, thereby causing the oscillatory force regime. Two characteristic layer thicknesses are detected: ${∆}_{1}$ ∼ 9.2 ± 1.1 Å (D ∼ 6 nm) and ${∆}_{2}$ ∼ 6.8 ± 1.1 Å (D ∼ 7.8 nm). Occasionally, more steps were detected at higher forces, F/R > 10 mN/m and ${∆}_{0}$ ∼ 6.1 to 7.4 Å (D ∼ 3.3 nm), and at lower forces, F/R < 0.2 mN/m and ${∆}_{3}$ ∼ 6.5 ± 0.4 Å (D ∼ 8.5 nm). The distribution of step sizes reflects the varying composition of the layers as a function of distance from the negatively charged mica surface. The overall decrease in size compared to the dry SiIL qualitatively agrees with the cluster size distribution predicted by MD simulations. Some layers are smaller than those measured in the pure IL (∼7.2 Å), which is consistent with the decrease in the length scales $l_{2}$ and $l_{3}$ measured by WAXS and with the presence of Na^+^ and/or water molecules in these layers.

The equilibrium surface forces in water-in-SiILs with $x_{w}$ ∼ 0.3 are highly reminiscent of the previous case and are summarized in Fig. 2 (G and H). Increasing the water content to $x_{w}$ ∼ 0.3 maintains the quasi-exponentially decaying force, with a similar decay length (d = 6.8 ± 1.4 nm across separate experiments). The major difference is that the magnitude of the force is larger, with corresponding larger onset separation. Several layers are squeezed out at separations smaller than ~8 nm, with an average size of ∆ ∼ 6.65 ± 0.1 Å. At F/R ∼ 20 mN/m, the layer thickness slightly increases to ${∆}_{0}$ ∼ 7.0 ± 1 Å. The larger step size at $x_{w}$ ∼ 0.1 (${∆}_{1}$ ∼ 9.2 ± 1.1 Å) is missing at $x_{w}$ ∼ 0.3, which is consistent with the smaller cluster sizes observed in the simulations. The higher water content also leads to a shift of the force walls to smaller surface separations, $D_{0}$ ∼ 3 nm, when compared to the water-in-SiIL with $x_{w}$ ∼ 0.1 ($D_{0}$ ∼ 5 nm at F/R ∼ 25 mN/m). This indicates that there is a thinner irremovable film at the closest approach, implying a different near-surface composition. A similar shift of the force wall was observed for [EMIM][TFSI] equilibrated at 15 and 35% RH (corresponding to water mole fractions of 0.013 and 0.03, respectively) (*39*). That study further showed that when [EMIM][TFSI] confined between mica surfaces (D ∼ 2 to 3 nm) is exposed to humid air, ion layers are spontaneously exchanged by water molecules. This ion-water exchange reduces the repulsive interaction between the surfaces, thereby explaining the observed shift of the hard wall, possibly because water partially screens the electrostatic interaction between ions and reduces overscreening (*39*). Once again, upon retraction of the surfaces, there is a measurable pull-off force (Fig. 2H).

Concurrently with the measurement of the force-distance curves, the SFA also measures the refractive index of the liquid confined between the two mica surfaces in real time as a function of separation, i.e., n(D). The refractive index is a measure of the density of the confined liquid, and hence, changes in structure and/or composition influence its value. One can expect an error in refractive index smaller than ∼0.01 in the range of D ∼ 10 to 100 nm for ultraprecise D-measurements (error, ±25 pm) (*40*). In this D-range, the average refractive index of the neat IL is 1.43 (fig. S8A), which is very close to the reported bulk refractive index (1.423) (*41*). The refractive index of the dry SiIL at D ∼ 100 nm is smaller, $n_{\text{SiIL}}$ ∼ 1.41, indicating that the addition of sodium reduces the refractive index. At large D values, the refractive index of the water-in-SiIL with $x_{w}$ ∼ 0.1 remains unchanged within the measurement precision (∼0.01) (fig. S8, B to D), but an increase in the water content to $x_{w}$ ∼ 0.3 leads to another small but noticeable decrease in the average refractive index to ∼1.405.

The refractive index of the neat IL remains constant over the entire D-range that permits reliable measurements, indicating that the composition under confinement is essentially the same as in the bulk. For both dry and humid SiILs, however, the average refractive index value decreases with decreasing surface separation, which indicates a change in composition—either intrinsic to the surface layers or as a result of confinement. In the case of the dry SiIL, there is a drop in refractive index at D < 30 nm (before the transition), potentially caused by the increase in sodium concentration. After the structural transition in the dry SiIL, a further drop in refractive index is observed.

The addition of water leads to a more substantial drop in refractive index with a decrease in D and more so for $x_{w}$ ∼ 0.3 compared to $x_{w}$ ∼ 0.1. The refractive index n of the water-in-SiILs can be approximately estimated from (*42*)

$$n=n_{\text{SIL}}-\phi \left(n_{\text{SIL}}-n_{w}\right)$$

where ϕ is the volume fraction of water, $n_{\text{SiIL}}$ is the measured refractive index of the dry SiIL at each value of D, and $n_{w}$ = 1.33 is the refractive index of water. Taking D ∼ 30 nm and $x_{w}$ = 0.3, this estimation leads to a volume fraction of water of ∼8 ± 7 vol %, which increases to ∼33 ± 7 vol % when the electrolyte is confined at D ∼ 15 nm. In the case of water-in-SiIL with $x_{w}$ = 0.1, a change in refractive index is only detected at D < 15 nm, yielding a water content of ∼15 ± 7 vol %. Although these values are rough estimates because of measurement noise and the simplified model (e.g., the difference in sodium enrichment near the surface in dry and humid SiILs has been ignored), they support the contention that water is enriched near the mica surface. There is more water in the case of $x_{w}$ = 0.3, but in each case, the enrichment is ∼10× to 20× that of the bulk composition.

Surface force measurements in another SiIL, sodium bis(fluorosulfonyl)imide (NaFSI) in 1-ethyl-3-methylimidazolium bis(fluorosulfonyl)imide ([EMIM][FSI]) at $x_{s}$ ∼ 0.2, lead to force-distance curves similar to those measured in neat ILs (fig. S9). The different behavior of this SiIL is tentatively attributed to weaker ion-ion correlations with the FSI anion (*43*). Few reported experimental studies have revealed ion-specific effects on the interfacial structure of SiILs with lithium-based chemistries. For instance, surface force balance measurements have demonstrated the effect of lithium bis(trifluoromethanesulfonyl)imide (LiTFSI) on the structure of two imidazolium-based ILs at the interface with mica (*44*). In [EMIM][TFSI], LiTFSI clusters can closely approach the mica surface, while the adsorption of Li^+^ only is inhibited, even at concentrations as high as 20 mol % LiTFSI (*44*). Conversely, in [EMIM][FSI], no clusters were detected in the near-surface region; instead, Li^+^ ions adsorbed directly onto the mica surface (*44*). Atomistic simulations suggest that strong interactions between Li^+^ ions and the anions in the SiIL can prevent Li^+^ from directly adsorbing onto negatively charged surfaces (*45*). Consequently, the substantial effect of water on the surface forces of the TFSI-SiIL may be associated with the weakening of Na-TFSI ion-ion correlations by water (*12*, *33*).

### Interfacial structure of SiILs

**Fig. 3.**
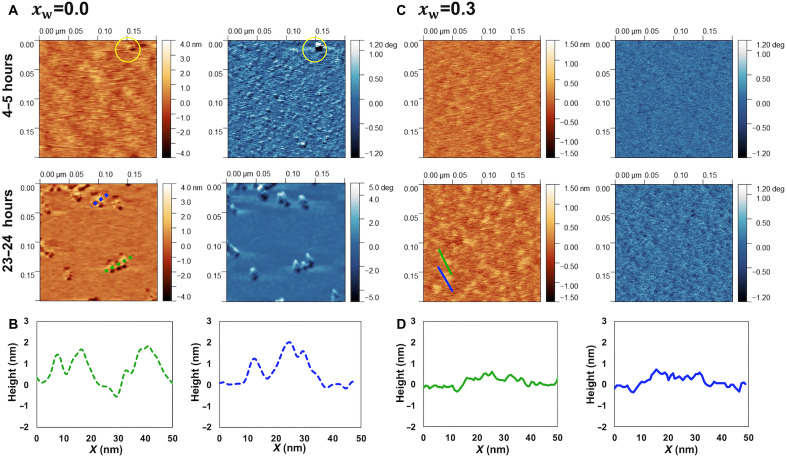
Tapping mode AFM images and cross sections in dry SiIL and water-in-SiIL. Tapping mode AFM images of topography (brown) and phase (blue) of mica immersed in (**A**) dry SiIL with $x_{s}$ = 0.10 NaTFSI. After 4 to 5 hours, the clusters become more prominent in the phase images. In this image, the largest cluster is highlighted with a circle, but note that smaller clusters are also present across the surface. The clusters continue growing over the next hours. (**B**) Representative cross sections of the aggregates in (A). (**C**) SiIL with $x_{s}$ = 0.10 NaTFSI equilibrated at 33% RH ($x_{w}$ = 0.3) at room temperature. (**D**) Representative cross sections of the clusters shown in (C). Each image displayed here was acquired after two prior scans of the same area; an example is shown in fig. S10. The persistence of clusters after consecutive imaging indicates that they were not removed by the tip during repeated scanning.

Figure 3A and fig. S10 show tapping mode atomic force microscopy (AFM) images of mica immersed in the dry SiIL with $x_{s}$ = 0.10 NaTFSI at room temperature and maintained in equilibrium with dry N_2_. Each image displayed in Fig. 3A was acquired after two prior scans of the same area; this is illustrated in fig. S10. The persistence of clusters in consecutive images indicates that they were not removed from the surface by the AFM tip during repeated scanning. After 4 to 5 hours of incubation with the dry SiIL, AFM images reveal a rough interfacial layer that cannot be brushed away by the AFM tip upon repeated scanning of the same area. The third image acquired at the same location (Fig. 3A) shows a few small clusters (∼10 nm long and 1 to 2 nm high). Following 23 to 24 hours of incubation with the dry SiIL, obvious aggregates (∼20 nm long and 2 to 7 nm high) are visible on the mica surface (fig. S10), and similarly, the AFM tip is unable to remove them upon repeated imaging (see also the third scan in Fig. 3A).

Representative cross sections are displayed in Fig. 3B. Relative to 4 to 5 hours of incubation, longer equilibration times lead to an increased number and size of aggregates, indicating that nucleation and growth continue over time. Together, these images support the conclusion that surface-promoted aggregation manifests upon equilibration of single, freshly cleaved mica sheets with the dry SiIL, with longer equilibration times enhancing this process. In addition, the accumulation of these aggregates between the mica surfaces provides a plausible explanation for the observed shift of the hard wall of the force in SFA experiments (Fig. 2, C and D).

Figure 3C shows topographic and phase images of the mica surface immersed in the water-in-SiIL with $x_{w}$ = 0.3. In contrast to the results for the dry SiIL, the surface remained free of aggregates, even after equilibration for 24 hours (see also fig. S11 at higher magnification). This confirms that the presence of water in these systems hinders the adsorption of clusters and further aggregation on the mica surface, consistent with SFA experiments under the same humidity condition.

**Fig. 4.**
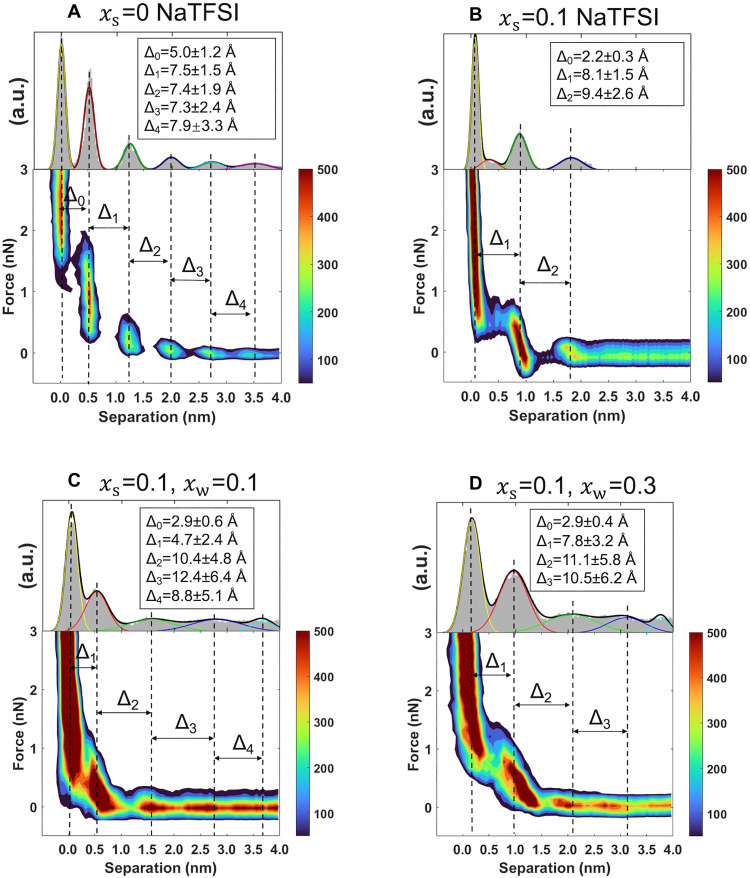
Interfacial structure of the IL, dry SiIL, and water-in-SiILs. AFM force-distance curves in electrolytes with (**A**) $x_{s}$ = 0 and (**B**) $x_{s}$ = 0.10 NaTFSI in equilibrium with (**C**) 11% RH and (**D**) 33% RH. The maximum applied force values were ∼12 nN [(A) and (B)] and 15 nN [(C) and (D)]. The mica surface was equilibrated for at least 1 hour with the electrolyte before measurements started. Each plot shows a heatmap constructed with 32 [in (A)], 63 [in (B)], 46 [in (C)], and 61 [in (D)] force-distance curves. Results were reproducible in other spots of the mica surface. The dimension of IL cation is 4 Å by 7.5 Å by 1.7 Å, and that of IL anion 3 Å by 7.6 Å by 2.5 Å (see the molecular structure of the IL in fig. S12). (A and B) Adapted with permission from Zhang *et al.* (*18*) (https://pubs.acs.org/doi/full/10.1021/acsnano.4c09355). Copyright 2024 Zhang *et al*. Published by the American Chemical Society under a CC-BY-NC-ND 4.0 license (https://creativecommons.org/licenses/by-nc-nd/4.0/deed.en). Table S2 shows the fitting parameters of the Gaussian distributions.

Given that SFA measurements do not resolve the electrolyte structure closest to the mica surface (D < 3 nm), additional force measurements were carried out by AFM using sharp Si tips. Figure 4 shows heatmaps constructed from at least 40 force-distance curves, each taken at a different location within an area of 500 nm by 500 nm (see the Supplementary Materials text). The discrete steps in the force-distance curves indicate the presence of multiple layers of ions/water near the mica surface (*46*). Histograms of the surface separation were constructed to determine the layer thickness (∆) and the standard deviation from Gaussian fits (see plots above the heatmaps and fitting parameters in table S2). For the neat IL ($x_{s}$ = 0), four layers with sizes of ${∆}_{0}$ ∼ 5.0 Å, ${∆}_{1}$ ∼ 7.5 Å, ${∆}_{2}$ ∼ 7.4 Å, ${∆}_{3}$ ∼ 7.3 Å, and ${∆}_{4}$ ∼ 7.9 Å were detected (Fig. 4A), consistent with an earlier study (*47*) and with SFA measurements; the corresponding standard deviation is shown in the plot. Considering the negative charge of the mica surface, the ${∆}_{0}$ value can be associated with a cation-rich ([EMIM]^+^) layer closest to the surface. The values of ${∆}_{1}-{∆}_{4}$ agree with the mean diameter of an ion pair, i.e., $a=\rho^{1/3}{\left(MN_{A}\right)}^{-1/3}$, with ρ being the IL density, M being the molar mass, and $N_{A}$ being the Avogadro number. The anion and cation are of similar size (fig. S12), and therefore, it is not possible to distinguish between charge-neutral layers and anion or cation enrichment beyond the first layer.

Adding salt at $x_{s}$ = 0.10 notably modified the interfacial structure. Only two layers were clearly observed in the heatmaps in the same range of forces as for the dry IL (Fig. 4B); however, a third layer was often resolved at a higher yet varying force on the individual force profiles with ${∆}_{0}$ = 2.2 ± 0.3 Å (fig. S13A). This suggests that Na^+^ is present on the mica surface. Previous AFM studies of mica in propylammonium nitrate with ∼60 mM alkali metal salts also suggested that the metal cations preferentially adsorb to negatively charged sites on the mica surface despite their much lower concentration compared to the IL cation (*48*). The varying force required to push through this layer, the small size of this layer (${∆}_{0}$) compared to the thickness of the hard wall in the heatmap, and the fact that it was not measured in all force-distance curves would preclude such a layer from being resolved in the heatmap. The thicknesses of the following near-surface layers are ${∆}_{1}$ = 8.1 ± 1.5 Å and ${∆}_{2}$ = 9.4 ± 2.6 Å, i.e., larger than those in the neat IL. This suggests that small ionic clusters are arranged as layers close to the surface.

The force-distance curves in the water-in-SiIL with $x_{w}$ = 0.1 reveal four interfacial layers, with ${∆}_{1}$ = 4.7 ± 2.4 Å, ${∆}_{2}$ = 10.4 ± 4.8 Å, ${∆}_{3}$ = 12.4 ± 6.4 Å, and ${∆}_{4}$ = 8.8 ± 5.1 Å (see Fig. 4C). For the water-in-SiIL with $x_{w}$ = 0.3, three layers were measured, and their sizes are ${∆}_{1}$ = 7.8 ± 3.2 Å, ${∆}_{2}$ = 11.1 ± 5.8 Å, and ${∆}_{3}$ = 10.5 ± 6.2 Å. An additional layer was often measured at variable higher force in both water-in-SiILs, with ${∆}_{0}$ = 2.9 Å (fig. S13, B and C).

Overall, the addition of water causes more diffuse peaks in the force-distance curve (note the large standard deviation of Δ), suggesting a more disordered electrolyte interfacial structure. The thickness of the innermost surface layer (${∆}_{0}$ ) is larger than in the dry SiIL. Hydrated Na^+^, being kosmotropic, is expected closest to the surface-adsorbed water. The different value of ${∆}_{1}-{∆}_{3}$ in the two water-in-SiILs indicates different interfacial compositions, likely due to the greater water content at 33% RH, as inferred from refractive index measurements. Note that an increase in layer thickness was also reported for [EMIM][TFSI] and other imidazolium ILs at small separations from the mica surface when equilibrated at 33% RH because of the presence of water (*39*).

Notably, the layer thickness ${∆}_{2}$ − ${∆}_{3}$ in both water-in-SiILs is larger than inferred from SFA experiments, yet they are resolved at smaller separations from the surface in AFM. It is important to emphasize that the AFM tip is sensitive to individual aggregates and clusters, whereas the SFA measures the average over a few square micrometers. SFA measurements of cyclohexane on mica contaminated with nanoparticles (*49*) showed weaker layering forces and smaller layer thicknesses compared to particle-free mica. This behavior was attributed to the increased disorder within the layers (defects). Similarly, previous AFM work showed that larger tip radii resulted in smaller layering forces, which was proposed to arise from the intrinsically higher roughness of larger tips and enhanced local disorder at asperities (*50*). Such implicit disorder in water-in-SiILs could thus result in an overall smaller layer thickness measured by SFA. Nonetheless, it is reasonable that clusters of different compositions and, thus, different sizes are located at different distances from the mica surface, leading to the different characteristic sizes measured by AFM and SFA.

### Electrochemical properties of SiILs

The change of the structure upon addition of water also influences the electrochemical properties of this SiIL. The conductivity varies with water content. For the IL, it is 4.70 mS/cm at 22.5°C, but it drops to 2.52 mS/cm in the dry SiIL, consistent with the increase in viscosity (*18*). However, adding water to the SiIL increases conductivity—initially only slightly (2.97 mS/cm for $x_{w}$ = 0.1) and then more substantially, reaching 5.56 mS/cm for $x_{w}$ = 0.3, surpassing the conductivity of the neat IL.

**Fig. 5.**
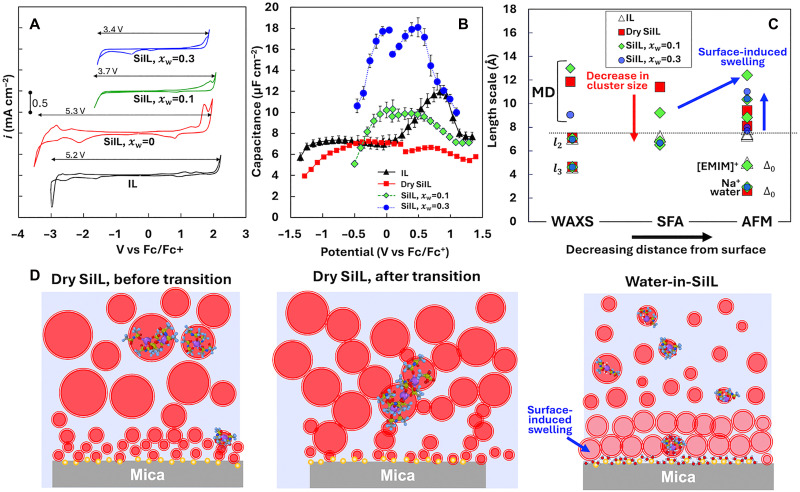
Electrochemical properties and nanostructure. (**A**) Cyclic voltammograms and (**B**) differential capacitance of dry IL ($x_{s}$ = 0) and $x_{s}$ = 0.1 NaTFSI in [EMIM][TFSI] and water-in-SiILs with water contents $x_{w}$ = 0.1, and $x_{w}$ = 0.3. Gold was used as the working electrode for these measurements. (**C**) Summary of the nanostructure from WAXS, MD simulations, SFA, and AFM measurements. The size shown to represent the clusters inferred from the MD simulations (above the dashed line) corresponds to that of the most frequent cluster. (**D**) Cartoon representing the transition of the nanostructure of IL, dry SiIL, and water-in-SiILs from bulk to the surface. For water-in-SiILs and neat IL, SFA and AFM resolve a layer thickness similar to the correlation length between ions of same charge ($l_{2}$). In the case of dry SiIL, layers larger than an ion pair are detected by SFA, reminiscent of the bulk nanostructure inferred from MD simulations. Layers become a bit smaller closer to the surface, as revealed by AFM. AFM reveals surface-adsorbed layers of the size of single ions (EMIM^+^ and Na^+^) in the IL and dry SiIL. Water reduces the size of the clusters in bulk (MD) and resolved by SFA (see the red arrow) but swells the layers closer to the mica surface (see blue arrow), except for the surface-adsorbed species, which are of the size of single ions and water molecules.

Cyclic voltammetry (CV) and electrochemical impedance spectroscopy (EIS) were carried out on gold as the working electrode. The electrochemical stability window (ESW) of the two water-in-SiILs is smaller than that of the dry SiIL, which is ∼5.3 V—slightly larger than that of the dry IL (Fig. 5A). Note that despite the water content, the ESW is >3 V for the two water-in-SiILs. Linear sweep voltammetry (LSV) shows consistent results (fig. S15). Together with the AFM and SFA measurements, these results support the presence of water near the negatively charged surface, although with reduced activity compared to free water—possibly due to interactions with Na^+^ (*12*).

The double-layer capacitance was obtained from fitting an electric circuit model to the EIS spectra (see fig. S16). The differential capacitance in the IL ($x_{s}$ = 0) shows a distinct camel-shaped curve with a more prominent peak at positive potential, which is consistent with the result for the same IL on glassy carbon electrodes that has been reported by Klein *et al.* (*51*) (see Fig. 5B). The dry SiIL exhibits a slightly asymmetric camel-shape differential capacitance, with a value of ∼7 μF/cm^2^ close to 0 V versus ferrocene/ferrocenium (Fc/Fc^+^) redox couple. The magnitude of the peak at positive potentials drops substantially compared to the neat IL, and the potential of the local maxima also shifts to smaller potentials. The change is much less prominent at negative potential. Notably, the differential capacitance of the water-in-SiIL with $x_{w}$ = 0.3 is much greater in the potential range of ±1 V (∼18 μF/cm^2^ at 0 V versus Fc/Fc^+^), which points at the improved screening capability of the water-in-SiIL compared to the dry SiIL and the neat IL. Furthermore, the camel shape is somewhat less prominent, as if the differential capacitance were transitioning toward a bell shape; this is also true for the water-in-SiIL with $x_{w}$ = 0.1.

Similar capacitance results were obtained from a more precise method to analyze EIS data (see fig. S17). This method accounts for a distribution of diffusion times through the double layer, arising from diffusive rearrangements of the ionic clusters in the electrical double layer (EDL), which more precisely reflects the electrolyte behavior investigated here.

## DISCUSSION

We have shown that surface forces for the investigated water-in-SiILs with the [TFSI]^−^ anion are similar to those measured for the neat IL: (i) The force decays quasi-exponentially with a decay length of d ∼ 6 to 7 nm, (ii) there is good agreement between subsequent force-distance curves upon approach, and (iii) a short-range oscillatory (structural) force reflects the layered arrangement of the ions/water with a varying step size depending on the water content and the distance from the surface. (iv) These features are maintained upon an increase in the mole fraction of water from $x_{w}$ ∼ 0.1 to 0.3, but the hard wall shifts to smaller separations, and the layer size distribution changes. Along with the decrease in the refractive index and the interfacial structure resolved by AFM, these results reflect the enrichment of water near the mica surface. (v) Furthermore, there is no evidence for the onset of a structural transition during the measurements, differing substantially from the behavior of the dry SiIL.

The large decay length of the surface force (d) deserves discussion. At sufficiently high concentrations—after the so-called Kirkwood transition—the electrostatic interaction in concentrated electrolytes follows damped oscillations (*22*). The decay length of these damped oscillations is larger than the Debye length $\lambda_{D}$ (typically up to ∼5 to 10× $\lambda_{D}$), and it increases with concentration, leading to the concept of underscreening. Anomalous underscreening refers to the experimentally observed decay length of the exponential (monotonic) long-range force, as measured by SFA, which can be up to ∼100× $\lambda_{D}$ (*21*, *52*). Lee and Perkin’s analysis led to $\lambda_{s}∼4\pi l_{B}c_{\text{ion}}a^{3}$, where a is the mean ion diameter, $l_{B}=q^{2}{\left(4\pi \varepsilon \varepsilon_{0}k_{B}T\right)}^{-1}$ is the Bjerrum length, $c_{\text{ion}}$ is the ion concentration, $\varepsilon \varepsilon_{0}$ is the dielectric constant of the medium, $k_{B}$ is the Boltzmann constant, and T is the temperature; note that previous study (*21*) used Gaussian units for the Bjerrum length. Anomalous underscreening is often described by a scaling law $\frac{\lambda_{s}}{\lambda_{D}}={\left(\frac{a}{\lambda_{D}}\right)}^{n}$ for $\frac{a}{\lambda_{D}}>1$, with n = 3. So far, theory and simulations typically predict exponents n = 1 to 2 (*17*, *53*), although values between 2 and 3 have been reported from simulations of surface forces (*54*).

We have tested the applicability of this model to the electrolytes studied here (see the Supplementary Materials text for details). Lee and Perkin (*21*, *52*) proposed to estimate the mean ion diameter a of ILs using density and ion pair concentration to calculate the molecular volume (methods 1 and 2 in table S3) and the correlation length $l_{3}$ corresponding to the adjacency peak $q_{3}$, which they proposed to be more precise (method 3 in table S3). We found that only one of the density-based methods matches well with the previously reported a value for [EMIM][TFSI] (*52*). It is unclear how to define an ion pair for a mixture of NaTFSI and [EMIM][TFSI] and whether hydration (in the water-in-SiILs) can additionally influence the mean ion diameter. Furthermore, determining an effective size for the SiILs—where clusters are present—could be more complex than these simplified approaches suggest. All the approximations made to determine a for the SiILs are described in the Supplementary Materials text. Figure S18 compares calculated $\lambda_{s}$ values on the basis of anomalous underscreening and d from SFA force measurements. The fits to $d/\lambda_{D}$ for the IL and water-in-SiILs yield power exponents n equal to 2.6, 2.4, and 2.1 on the basis of methods 1, 2, and 3, respectively, which not only reveals that anomalous underscreening cannot be universally applied to all highly concentrated electrolytes but also raises the question on how to robustly define the ion diameter in this model.

The absence of long-range forces in AFM measurements (Fig. 4), consistent with previous studies on highly concentrated electrolytes (*17*, *53*), and our predicted screening lengths for ILs (subangstrom) (*54*, *55*) are noteworthy. Because ILs are viscous, the hydrodynamic drag during squeeze-out of the confined electrolyte in SFA measurements can be substantial (*24*, *56*) (see also calculations in the Supplementary Materials text). Nonetheless, the SFA force determination relies on a baseline correction that substantially reduces viscous contributions in the range of velocities used here (*36*). However, the uncertainty of this approximation increases with decreasing surface separation and more so if confinement-induced slowing of ion relaxation becomes relevant (*39*, *57*). In addition, simulations of ion flux through biological membrane channels have shown that violation of electroneutrality can generate long-range electric fields that drive three-dimensional ionic reorganization (*58*). While dynamic effects associated with local electroneutrality breakdown may arise, the contribution of such phenomena to SFA measurements remains to be elucidated. Notably, recent surface force measurements using two different SFA setups have shown that substantially longer equilibration times are required to eliminate dynamic effects in ILs (*24*). So-called quasi-static SFA measurements (performed at approach velocities an order of magnitude lower than those used here) yield short-range forces in [EMIM][TFSI], with a decay length of d ∼ 1 nm. On the basis of those results, the screening length is much smaller than the decay length of the force shown in Fig. 2A. It is reasonable to expect that hydrodynamic effects also influence SFA measurements in SiILs (Fig. 2, C to H).

Notably, MD simulations predict an oscillatory, decaying electrostatic force in highly concentrated electrolytes when ions transition from local electroneutrality to a regime characterized by periodic charge-density oscillations (*54*, *55*). In contrast, SFA measurements in highly concentrated electrolytes show a monotonically decaying force with increasing surface separation (beyond the layering region), even under quasi-static conditions (*24*). In (*24*), the absence of oscillations was attributed to $d/a≃\lambda_{D}/a$ remaining below the Fisher-Widom threshold of ∼2.45. However, we cannot exclude that residual dynamic effects suppress such oscillations.

Our findings—including evidence of a structural transition in the dry SiIL as well as the differential capacitance—suggest that the mechanism underlying the long-range force behavior of dry and water-in-SiILs cannot be captured by electrostatic arguments. Consistent with this, theory and MD simulations predict a screening length below 0.1 nm for the dry SiIL (*18*, *59*), in part due to the multivalency of ionic clusters, which increases the effective ionic strength. In addition to the described dynamic effects, dispersion interactions between ions and surfaces have been suggested to influence the decay length of surface forces in highly concentrated electrolytes (*53*). In ILs, dispersion interactions typically contribute 8 to 15% of the total interaction energy (*53*) and even more (12 to 16%) for imidazolium-based ILs (*60*). However, the magnitude of dispersion interactions is smaller, and their decay is faster than that of Coulombic interactions (i.e., 1/*r*^6^ versus 1/*r*, respectively), and therefore, equilibrium surface forces are still expected to be dominated by electrostatics. Nevertheless, Kjellander and Ramirez (*61*) have proposed that coupling between electrostatic and dispersion interactions exists and that this coupling can alter the form of the decay. Furthermore, the cluster-like nanostructure of the electrolyte should influence this coupling and the resulting force profile. In addition, ion-dispersion forces could be influenced by the presence of water-in-SiILs (*62*). An early study by Parsons and Ninham (*62*) has shown how dispersion forces can explain phenomena such as secondary hydration forces in aqueous electrolytes. A surface hydration layer introduces an excluded volume that chaotropic ions like EMIM^+^ (*63*) cannot penetrate, whereas kosmotropic ions (e.g., Na^+^ and Ca^2+^) can. As the surface separation decreases, kosmotropic counterions remain confined, leading to an increase in the local concentration. While the resulting entropic repulsion gives rise to a short-range, exponentially decaying repulsive force, ion-surface dispersion forces lead to a second exponentially decaying force, which is referred to as secondary hydration force and is of longer range (e.g., d ∼ 3.0 nm for 5 M CaCl_2_) (*62*). These effects have yet to be fully incorporated into theoretical and simulation frameworks for modeling surface forces. We maintain that capturing the correct nanostructure of the electrolyte, nonlocal nonlinear dielectric polarization, and dynamic effects—both in the bulk and near the surface—is critical for understanding screening and surface interactions.

The effect of water on the SiIL nanostructure is summarized in Fig. 5C. The plot shows the characteristic length scales determined by WAXS for the anion-cation ($l_{3}$) and cation-cation ($l_{2}$) distances, both of which are slightly influenced by the presence of Na salt and water. In pure ILs, these length scales align well with the layer thicknesses measured via force-distance measurements: $l_{2}$ (distance between like-charged ions) corresponds to the value of $\Delta_{1}-\Delta_{4}$, while $l_{3}$ matches the thickness of the surface-adsorbed layer of EMIM^+^ cations ($\Delta_{0}$). In the dry SiIL, MD simulations, SFA, and AFM reveal clusters that are larger than the typical cation-cation distance ($l_{2}$, dashed line), except near the mica surface, where Na^+^ adsorption occurs ($\Delta_{0}$). These clusters decrease in size closer to the surface, indicating surface-induced changes in composition—a behavior also observed in Li-WiSE systems (*64*).

Water ($x_{w}$ = 0.1 and $x_{w}$ = 0.3) introduces disorder, and the overall cluster size decreases in both bulk (simulations) and confinement (SFA). Notably, AFM force-distance curves detect a broad layer size distribution closest to the surface. While clusters of similar size to those in the dry SiIL persist, larger clusters are also present. This “swelling” of the clusters is attributed to a higher water concentration near the hydrophilic mica surface compared to the bulk. We thus posit that surface-adsorbed ion layers and clusters carry more water than in the bulk, which is supported by the measurements of the refractive index. The clusters in the water-in-SiIL with $x_{w}$ = 0.1 are larger than those at $x_{w}$ = 0.3, potentially because of the larger number of anions/Na^+^ in these clusters. The enrichment of water near the surface is consistent with the smaller ESW of the water-in-SiILs resulting from the hydrogen evolution reaction.

While a dielectric enhancement of the capacitance is expected upon addition of water (*65*), the transition to a bell-shaped profile indicates a fundamental change of the EDL in water-in-SiILs. Notably, neutral “tails” in IL ions give rise to the camel shape, as they provide extra degrees of freedom for the field-induced charge rearrangements (*66*). Accordingly, increasing water content reduces the ion concentration, which by itself should favor a camel-shaped capacitance. However, this effect is outweighed by another competing mechanism. As cation-anion interactions weaken in the presence of water (*12*, *33*), denser packing of ions within the EDL may occur, resulting in a higher occupied-volume fraction (reduced void fraction) and decreased compressibility, thereby promoting a bell-shaped capacitance profile (*67*, *68*). In addition, reduced electrostatic correlations within ionic clusters may promote the expulsion of co-ions (TFSI^−^) from the interfacial region while enhancing surface charge screening by Na^+^ counterions. This redistribution thus increases the capacitance, as well.

The results presented here have important implications for the application of these electrolytes in batteries. SEI formation is governed by the interfacial structure and the charge-screening properties of the EDL and is therefore influenced by the interfacial ionic aggregation characteristic of SiILs. Our ongoing studies show that the surface-enhanced aggregation observed for the dry SiIL on the negatively charged mica surface directly relates to the SEI precursor on the anode surface. Notably, this surface-enhanced aggregation is suppressed in the two water-in-SiIL systems studied here. Consistent with this, a previous study on a Na-based SiIL containing 50 mol % NaFSI in *N*-methyl-*N*-propylpyrrolidinium bis(fluorosulfonyl)imide demonstrated that the presence of water (up to 0.5 wt %) promoted the formation of a more stable and uniform SEI while suppressing dendritic growth (*26*). Together, these findings lead us to propose that SEI nucleation can be tuned through the partial substitution of the IL with water, offering a practical strategy to control SEI growth—potentially slowing it—and to tailor the SEI structure toward a more compact, homogeneous, and stable interphase.

In conclusion, this study demonstrates that the nanostructure and interfacial behavior of SiILs are highly sensitive to water content and to the proximity to a charged surface. Under dry conditions, SiILs form polyionic clusters that give rise to long-range, non-Debye surface forces, driven by confinement-induced ordering, surface-promoted aggregation, and dynamic effects. The addition of water disrupts these clusters and reduces their size and order, leading to surface forces that more closely resemble those of neat ILs. AFM and SFA measurements confirm that hydration eliminates the structural transition observed in dry systems and leads to more diffuse, layered interfacial structures. Refractive index data and AFM imaging further show that water preferentially enriches near hydrophilic surfaces, altering local composition. Despite this, the ESW remains wide (∼3 V). Nonetheless, the transition from camel- to close-to-bell-shaped differential capacitance profiles with increasing water content indicates a fundamental change in the EDL because of weakened ion-ion interactions by water, with an overall improved screening behavior. These findings highlight the limitations of classical screening models, i.e., the Gouy-Chapman model, and underscore the importance of accounting for an interfacial nanostructure when designing next-generation electrolytes for electrochemical applications. In addition, understanding the interfacial structure will improve our ability to control the formation of the SEI. Future work will focus on this aspect, along with the role of ion-specific interactions.

## MATERIALS AND METHODS

### SiIL structure from MD simulations and WAXS

NaTFSI (97%, RoCo, and 99.5%, Solvionic), NaFSI (99.9%, Solvionic), and the ILs [EMIM][TFSI] (97%, Sigma-Aldrich) and [EMIM][FSI] (99.9%, Solvionic) were dried at 50°C under vacuum in a Thermo Fisher Scientific Isotemp vacuum oven for more than 48 hours. The SiIL samples were prepared under a N_2_ atmosphere with a mole fraction ($x_{s}$) of 0.10 of NaTFSI in [EMIM][TFSI]. They were mixed in a Vortex-Genie 2 mixer for more than 1 min and then sonicated in a VWR Symphony Ultrasonic Cleaner for 90 min at 50°C. The water content was determined by Karl Fischer after electrochemical characterization on four different samples, and it is 137 ± 50 parts per million (ppm), which is taken as the water content in the dry SiIL. SiIL samples were stored in containers of supersaturated LiCl and Mg(Cl)_2_ solutions. The water content was measured gravimetrically to determine when equilibrium was achieved and then rigorously determined using Karl Fischer coulometric titration. This led to a mole fraction of water $x_{w}$ equal to 0.110 ± 0.002 (5793 ± 128 ppm) and 0.289 ± 0.002 (18,819 ± 213 ppm), respectively. The solutions were investigated within 2 weeks after preparation. The dry SiIL was slightly turbid because of the presence of ∼2-μm droplets in solution (<0.8 vol %), indicating a liquid-liquid phase separation (fig. S14). After filtering the dry SiIL, the droplets were removed. The water-in-SiILs were clear, and hence, water hinders this minor phase separation.

### Wide-angle x-ray scattering

IL and SiIL samples were transferred into 1.0-mm glass number 50 borosilicate capillaries (Hampton Research, US) in a glove box that was purged with N_2_. The capillaries were then sealed with hot melt adhesive. The WAXS experiments were performed using a SAXSLabs Ganesha x-ray scattering instrument (Xenox) composed of a Cu 50-kV, 1.54-Å Xenocs Genix ultralow divergence SL X-ray Source. The sample-to-detector distance was 100 mm, and each sample was measured over a 15-min period. This yields a *q* range of 0.7 to 27 nm^−1^ (equivalent to 9.0 to 0.23 nm in real space). The two-dimensional diffraction data were azimuthally averaged upon acquisition on a 170-μm pixel–spaced, single-photon counting Dectris Pilatus 300k 20-Hz Detector, and the data integration was performed using FIT2D (European Synchrotron Radiation Facility) and Igor Pro version 8 software (WaveMetrics, US). Before each measurement, a blank transmission measurement was taken for background subtraction. The calibration of the sample-to-detector distance is carried out regularly and before each measurement to ensure accuracy.

### Surface forces apparatus

SFA force measurements were performed following well-established procedures (*20*). Details about accuracy, resolution, mechanical drift, thermal stability, and imaging of our extended SFA are described in detail in the literature (*40*, *69*). The statistical precision of the surface-separation measurement is ±25 pm over a distance between 0 and 10 μm, thanks to an automated simultaneous evaluation of multiple fringe wavelengths via a numerical method called fast spectral correlation (*40*).

A volume of ~500 μl of electrolyte sample was filtered through a 220-nm syringe filter and injected into the gap between two mica surfaces in the fluid cell. The SFA cell was purged with a gentle stream of dry N_2_ to prevent water uptake by the IL and the dry SiIL, while the temperature was maintained constant at 25 ± 0.02°C with a homebuilt temperature-control device (*69*). This method kept the RH smaller than 3% RH (labeled as “dry” condition). We estimated the water content in the SiILs at 3% RH through the interpolation of the water content at 0, 11, and 33% RH. This gives a maximum water content of 1696 ppm and an average value of ~1131 ppm (∼2% RH, $x_{w}$ = 0.02) in the dry SiIL, while for the neat IL, these values are much smaller (129 and 86 ppm, respectively). For the water-in-SiILs, the RH in the SFA cell was controlled at either 11 or 33% RH by mixing dry and water-saturated nitrogen to maintain the water content constant in the electrolyte.

In the SFA, the electrolyte sandwiched between the two mica surfaces was equilibrated for 12 hours while keeping the surfaces separated at 3 to 5 μm. Surface force measurements were then carried out at a constant velocity of 0.15 nm/s until a maximum force of ∼150 mN/m was achieved, after which the surfaces were separated to a distance of *D* ∼ 1 μm (force-distance curve FC1) and immediately approached at the same velocity to measure the following force-distance curves, and this was repeated to measure several force-distance curves (FC2 to FC5). During the measurements, the RH in the cell was constantly monitored with a sensor (Sensirion, Switzerland) placed ∼1 cm above the mica surfaces.

The measured forces F were normalized by the radius R of the undeformed mica surfaces, determined by lateral scans of 100 μm in *x* and *y* directions. This allows the forces to be related to the interaction energy via the Derjaguin approximation. However, it is inevitable that forces above 20 to 30 mN/m lead to an increase in the radius of curvature R resulting from the deformation of the glue underneath mica, which is ignored in the calculation of F/R values. Even though this results in an overestimation of surface forces, it is a common approach to plot the surface forces normalized by R. Furthermore, the absolute accuracy of D (e.g., absolute zero) deviates under high forces (*70*), and an error in D smaller than 1 nm has been estimated at F/R ∼ 250 mN/m as a result of mica compression (*71*). The greatest error in D would yield a maximum instrumental error in force of 0.1 mN/m. However, changes in film thickness at a given force (called film-thickness transitions of size ∆) are detected at full precision (*71*).

### Atomic force microscopy

Tapping mode AFM images were captured using a Cypher VRS AFM (Asylum Research, Oxford Instruments). Images with scan sizes of 500 nm by 500 nm, 200 nm by 200 nm, and 50 nm by 50 nm were taken at a scan rate of 8 Hz with soft cantilevers (0.09 ± 0.03 N/m, BL-AC40TS, Olympus) and a frame resolution of 256 by 256 pixels. At least three different areas were imaged on each sample, and each of them was imaged multiple times to examine changes over time. During measurements, the AFM cell was sealed and purged with humidity-controlled N_2_.

Force measurements were performed using a JPK (Santa Barbara, CA) AFM with CSC-37 silicon tips (MikroMasch, CA), with a nominal tip radius of ∼10 nm and spring constants of ∼0.3 to 0.8 N/m, as determined by the thermal noise method (*72*). The cantilevers were cleaned by ultraviolet ozone for 30 min before each measurement. A volume of ∼1 ml of IL/SiIL sample was filtered through a 220-nm syringe filter and injected into the AFM fluid cell, which was sealed and purged with humidity-controlled N_2_ during measurements. Force measurements were conducted on freshly cleaved mica surfaces after 1 hour of equilibration in the electrolyte, with maximum applied forces ranging between 12 and 15 nN in all replicate experiments. A total of 64 force curves were collected on an area of 500 nm by 500 nm in each force map, and at least two force maps were carried out per sample. A tip velocity of 20 nm/s was used for these measurements. Heatmaps shown in the manuscript are produced through a process of bivariate histograms, which convert raw scattered data points into a probability density plot (*73*). The specific choice of bin sizes determines the granularity of the map, effectively defining the resolution of the grid cells.

### Ionic conductivity

The conductivity of the electrolytes was measured using a Jenway Conductivity Meter 4510 equipped with a microvolume conductivity probe (4-mm diameter, 120 mm reach). The conductivity probe was mounted at the center of a polytetrafluoroethylene septum vial cap, and measurements were conducted with the sample vials fully sealed. Conductivity measurements were performed at a laboratory room temperature of 22.5°C.

### Electrochemical characterization

CV and LSV measurements were performed in a three-electrode cell configuration with a scan rate of 10 mV/s using a CHI700E potentiostat. Both LSVs (toward positive and negative potentials) started from a pristine working electrode. The working electrode is a 2-mm-diameter gold disk (CH Instrument Inc.) and polished with 0.05-μm alumina powder in deionized water slurry on a Microcloth polishing pad (Buehler), followed by sonicating in deionized water and ethanol for 15 min, respectively. The counter electrode and reference electrode are a gold wire (MSE Supplies) and a silver wire (Sigma-Aldrich), respectively, both cleaned by sonicating in ethanol. A volume of ∼0.5 ml of electrolyte was added to the cell to fully immerse the three electrodes.

EIS measurements were performed in the same three-electrode cell configuration using a Gamry Reference 620 potentiostat. The EIS spectra were measured with 10-mV ac voltage from 1 MHz to 0.1 Hz at each dc voltage. The sequence of the dc voltage always starts with the open circuit potential of the pristine system and then progresses toward the positive/negative limits (determined from CV) with a 0.1-V voltage step. After the measurement, ferrocene dissolved in the same SiIL was added to the cell, and cyclic voltammograms were recorded to determine the internal reference versus Fc/Fc^+^. The double-layer capacitance depicted in Fig. 5 was obtained from fitting an electric circuit model to the EIS spectra (see fig. S16). We also used a more precise method to evaluate the differential capacitance (see fig. S17). This method accounts for a distribution of diffusion times through the double layer, arising from diffusive rearrangements of the ionic clusters in the EDL. While the differential capacitance is similar to that obtained considering a constant phase element, this method provides a potential physical interpretation of constant phase element for the double-layer capacitance.

### MD simulations

We performed atomistic, classical MD of the studied electrolyte compositions using LAMMPS (*74*). For the dry SiIL, we included 100 Na^+^ cations, 900 [EMIM]^+^ cations, and 1000 [TFSI]^−^ anions. For the water-in-SiILs, we kept the number of ions, and included 111 and 430 water molecules for $x_{w}$ equal to 0.1 and 0.3, respectively. The water molecules were modeled with the extended simple point charge (SPC/E) force field. The Na^+^, [EMIM]^+^, and [TFSI]^−^ ions were modeled using the Canongia Lopes and Padua (CL&P) force field (*75*) but with the charges rescaled by 0.7 to take into account the nonpolarizable nature of the force field, which has been shown to improve the comparison to the experimental density of a similar SiIL (*9*, *37*).

The initial configurations and input files were generated with fftool and packmol (*76*). A time step of 0.5 fs was used with the velocity-Verlet integrator. Initially, the structures were relaxed, and then a temperature annealing cycle from 300 to 600 K and back to 300 K (where the temperature was ramped up by 150 K over 1 ns before being held there for another 1 ns, then again ramped up by 150 K over 1 ns and held for 1 ns, and then down, following the same method; with a 10-ns equilibration at 300 K thereafter) was performed in NPT (at 1 bar) to equilibrate the electrolyte. The Nosé-Hoover barostat and thermostat were used with 1000× and 100× the time step, respectively. The production run then collected 500 frames every 5 ps in the NVT ensemble at the equilibrated volume and 300 K. The density was determined. This led to densities of 1.49, 1.48, and 1.46 g/cm^3^ for the SiILs with $x_{w}$ equal to 0, 0.1, and 0.3, respectively. Note that the densities of the MD simulations are slightly below experimental values of dry SiILs (*5*, *28*), which could arise because of the simple charge rescaling used here (*28*, *29*).

From these sampled structures, we determined that a cutoff distance between Na-O, in both water and TFSI^−^ anions, of 3.65 Å gives the real-space criterion for determining whether Na^+^ was associated with anions or water. Using this real-space criterion, average coordination numbers were computed, and adjacency matrices were built between Na-TFSI to determine the aggregation in the electrolyte. From these adjacency matrices at each time step, we determined the number of aggregates containing l Na^+^ cations and m TFSI^−^ anions, $N_{\mathrm{lm}}$, and the number of associations in each of these aggregates. The dimensionless concentration in Fig. 1A was computed from the time average of $N_{\mathrm{lm}}$ over sampled structures, divided by the total number of lattice sites, $\Omega =N_{\text{Na}}+N_{\text{EMIM}}\xi_{\text{EMIM}}+N_{\text{TFSI}}\xi_{\text{TFSI}}+N_{W}\xi_{W}$, where each $\xi_{j}$ represents the number of lattice sites occupied by each species. Here, we take the lattice site equal to that of the Na^+^ cation, with water being approximated to the same size and [EMIM]^+^ and [TFSI]^−^ being approximated as seven times that size. Note that these values do not qualitatively change any results. The computed CBD, coordination number distributions, observed aggregates, and loops are shown in figs. S1 to S5.

## Supplementary Materials

### This PDF file includes:

Anomalous underscreening Hydrodynamic drag Figs. S1 to S18 Tables S1 to S3 References
