# Supplementary material for: Water doping sodium battery electrolyte controls nanostructure, interactions, and electrochemical properties
Source: Sci Adv. 2026 May 29;12(22):eaee3415. doi: 10.1126/sciadv.aee3415 (PMC13220881; doi:10.1126/sciadv.aee3415)
Supplement: Supplementary file 1 — Anomalous underscreening Hydrodynamic drag Figs. S1 to S18 Tables S1 to S3 References [file sciadv.aee3415_sm.pdf]

Science Advances
AAAS

Supplementary Materials for

Water doping sodium battery electrolyte controls nanostructure, interactions, and electrochemical properties

Xuhui Zhang *et al.*

Corresponding author: Zachary A. H. Goodwin, zac.goodwin@materials.ox.ac.uk;
Rosa M. Espinosa-Marzal, rosae@illinois.edu

Sci. Adv. **12**, eaee3415 (2026)
DOI: 10.1126/sciadv.aee3415

This PDF file includes:

Anomalous underscreening
Hydrodynamic drag
Figs. S1 to S18
Tables S1 to S3
References

Anomalous Underscreening model

The anomalous underscreening model states that the screening length λ_s is related to the ion diameter and Debye length through

$$\frac{\lambda_s}{\lambda_D} \propto \left(\frac{a}{\lambda_D}\right)^3$$

Eq. (S1)

Where a is the mean ion diameter and λ_D is the Debye length

$$\lambda_D = \left(\frac{\epsilon \epsilon_0 kT}{q^2 c_{ion}}\right)^{1/2}$$

Eq. (S2)

Where q=1.6.10^{-19} C, ϵ_0=8.85.10^{-12} C m^{-2}, k=1.38.10^{-23} J K^{-1}, T the temperature (K), c_{ion} is the ion concentration, and ϵ the relative permittivity. Note that all ions are monovalent, and therefore, the ion pair concentration, $c_{pair} = c_{ion}/2$. The anomalous screening model is expressed as (*21*):

$$\lambda_s {\sim} 4\pi l_B c_{ion} a^3$$

Eq. (S3)

Where l_B is the Bjerrum length:

$$l_B = \frac{q^2}{4\pi\epsilon\epsilon_0 kT}$$

Eq. (S4)

Note that when using Gaussian unit system for the Bjerrum length, like in (*21*), the 4π factor and ϵ_0 in Eqs. (S3)-(S4) are eliminated.

To directly compare the decay length of the surface force with the results from this model, we need to estimate the mean ion diameter a. We found three different methods that were employed in previous works to determine the mean ion diameter, which we compare next.

The first method (**method 1**) proposed by Lee et al.(*21*) is $a_1 = 1/2(V_{molec})^{1/3}$, where V_{molec} is the average molecular volume. V_{molec} is determined using the density of the electrolyte and the molar mass. However, the physical meaning of this equation is unclear. The second method (**method 2**) proposed by Smith et al. (*52*) estimates the mean ion diameter, a, from the cube root of the volume per ion pair halved, i.e. $a_2 = \left(\frac{V_{molec}}{2}\right)^{1/3}$.

To determine the ion diameter from **method 1** and **method 2**, we used the density of the electrolyte. The density of the pure IL is equal to 1.52 g/cm^3 from a prior experimental study.(*80*) M**ethod 1** leads to a_1=3.76 Å for [EMIM][TFSI] and agrees well with the reported value in (*52*) for this IL. Notably, this value aligns with some experimental observables. For example, AFM force measurements indicate that the thickness of a charged-neutral layer is Δ~7.5 Å, i.e. approximately twice the value of a_1.

For [EMIM][TFSI], **method 2** yields a value of 5.98 Å. Although this method was proposed by Smith et al. (*52*), the values reported in that same work (a_1=3.8 Å for [EMIM][TFSI]) do not agree with this result.

The third method (**method 3**) was also proposed by Smith et al. (*52*), and it states that the correlation length of the adjacency peak q_3 provides a more precise estimation of the mean ion diameter. The values $a_3 = l_3$ lie between the values calculated using **method 1** and **method 2** for the IL and the SiILs. For example, a_3=4.62 Å for [EMIM][TFSI].

As discussed above, there is uncertainty in determining the mean ion diameter of the neat IL. Additionally, it is unknown how to apply this method to determine the mean ion diameter for a mixture of two salts like NaTFSI and the IL. Another question is how to consider the effect of hydration. This question was already discussed by Smith et al. in ref. (*52*) for NaCl: hydration lead to the increase of the mean ion diameter from 2.94 to 5.2 Å, and led to a deviation of the calculated screening length from the anomalous underscreening prediction. Additionally, determining an effective ion size for the SiILs—where clusters are present—might be more complex than this simple approach suggests.

Without solving these uncertainties, we used the density of the dry SiIL to calculate a_1 and a_2 in the same way as for the IL, i.e. using the density reported in an experimental study.(*5*) Note that in this case, the layer thickness (Δ) obtained from AFM force measurements notably deviates from a_1 and a_2. Smith et al. (*52*) used the same mean ion diameter for the IL and for mixtures of IL and propylene carbonate. Similarly, we used the same values a_1 and a_2 for dry SiIL and water-in-SiILs, ignoring the effect of hydration. However, we used $a_3 = l_3$ from our WAXS measurements, and hence, different values were taken for SiIL and water-in-SiILs. The dielectric constant was taken as 12.3 (-) for the neat IL and the dry SiIL, and 12.8 (-) and 14.28 (-) for the water-in-SiIL with x_w=0.1 and 0.3, respectively, considering the volume % of water. The densities of the water-in-SiILs were determined by MD simulations to determine the molecular volume (see Materials and Methods for discussion on accuracy). The values of density, molecular volume, and c_{pair} used in the following calculations are shown in **Table S3**.

Finally, we used the ion diameters a_1, a_2 and a_3 of [EMIM][TFSI], dry SiILs and water-in-SiILs to determine the screening length λ_s using Eq. (S3). We also used a *modified* equation which yields better agreement with the experimental results:

$$\lambda_s' \sim 4\pi l_B c_{pair} a^3$$

Eq. (S5)

The only difference is that we substituted c_{ion} by c_{pair}.

If the ion diameter is taken to be a_1 (method 1)– the anomalous underscreening screening length resulting from this analysis $\lambda_{s,1} \sim 4\pi l_B c_{ion} a_1^3$ =14.32 nm, which remarkably deviates from the value reported of 7.1 nm(*52*) and from our measurements. However, using Eq. (S5) yields $\lambda_{s,1}' =$ 7.1 nm, and a good agreement with the experimentally determined decay length of the long-range forces (d) is found.

Using the mean ion diameter from method 2, a_2=5.98 Å, the calculated anomalous underscreening screening lengths for the IL are $\lambda_{s,2} \sim 4\pi l_B c_{ion} a^3$=57.3 nm and $\lambda_{s,2}'$==28.64 nm, which do not agree with the decay length of the long-range force of this IL. (*21, 52*).

The values for the mean ion diameter from **method 3**, $a_3 = l_3$ lie between the results from **method 1** and **method** 2. This method was used to determine the screening lengths $\lambda_{s,3}$ and $\lambda_{s,3}{}'$ according to the scaling analysis. The agreement with experiment is poor, with $\lambda_{s,3}$ =26.4 nm and $\lambda'_{s,3}$ =13.2 nm for the neat IL. The same methods were applied to calculate the λ_s-values of other electrolytes.

The λ_s- and $\lambda_s{}'$-values are shown as full and empty markers, respectively, in **Figure S18**. Overall, the $\lambda_{s,1}{}'$-values give the best agreement to the decay length of the exponentially decaying surface forces in [EMIM][TFSI] and in the water-in-SiILs; note that the experimental values (d) are 6.7, 6.1 and 6.2 nm, for IL, and the two water-in-SiILs, respectively, and the same values are plotted vs. $\frac{a_1}{\lambda_D}$, $\frac{a_2}{\lambda_D}$ and $\frac{a_3}{\lambda_D}$ (x-axis). A direct comparison to the dry SiIL is not possible since the force is not exponentially decaying and there is a very high variability; see also ref. (*18*).

The scaling model implies(*21*):

$$\frac{\lambda_s}{\lambda_D} = C\left(\frac{a}{\lambda_D}\right)^3$$

Eq. (S6)

With C=1 (*21, 52*), the transition to anomalous underscreening happens at $\frac{a}{\lambda_D}$=1. For the fits in **Figure S1**8, we thus chose C =1. In this case, the power exponents are 2.57 when using a_1, 2.30 when using a_2 and 2.05 when using a_3, and therefore, smaller than n=3. Exponents of up to 2 have been predicted by theory.(*55*)

Hydrodynamic drag

For the crossed-cylinder SFA geometry (two identical cylinders of radius R with perpendicular axes, equivalent to a sphere-on-plane configuration), the Reynolds (lubrication) equation gives the hydrodynamic force per unit radius as

$$F_{\mathrm{hyd}}/R \sim \frac{6\pi\eta UR}{D},$$

Eq. (S7)

where η is the viscosity (~35 mPa.s), U the approach velocity (0.15 nm s^{-1}), and D the surface separation. For [EMIM][TFSI], a simple estimate yields $F_{\mathrm{hyd}}/R = 2.47/D$ (with D in nm and F_{hyd}/R in mNm^{-1}). Thus, the hydrodynamic force is negligible at large separations (e.g., ~0.08 mN m^{-1} at $D = 30$nm, below the minimum force shown in Figure 2a), but increases as D decreases (e.g., 0.27 mN m^{-1} at $D = 10$ nm). For the dry SiIL, the viscosity is 59 mPa.s, and therefore, the hydrodynamic drag should be ~1.7× larger, if the other conditions would remain unchanged. Note that this factor cannot explain the change of the surface forces. The viscosity of the water-in-SiILs is not precisely known but it is smaller than that of the dry SiIL, and therefore, the hydrodynamic force is expected to be smaller.

Although there is hydrodynamic drag, SFA force determination relies on baseline correction that reduces viscous contributions. Specifically, the imposed motion of one lens is subtracted using a linear fit of the trajectory at large separations ($D \gtrsim 200$ nm).(*57*) Lhermerout and Perkin (*36*) reported that this standard procedure in dynamic force measurements removes viscous effects from the slope of the long-range force within the velocity range used here at sufficiently large separations ($D > 10$ nm). They showed that the decay length was not influenced by the hydrodynamic drag under these conditions. A more recent study has shown that the decay length is influenced by hydrodynamics, and the velocity of approach needs to be 10x smaller to effectively eliminate the viscous effects.

We note that recent MD simulations examined surface forces between two small, charged metal nanoparticles, explicitly accounting for hydrodynamic interactions as well as particle curvature and ion–ion interaction strength (*54*). These studies reported a power-law exponent n between 2 and 3, with values closer to 3 obtained for $l_B/a \sim 0.5$–2 and the smallest particle size ($R = 7.5a/2$). Our electrolytes yield l_B/a ~10-12 (assuming the bulk dielectric constant) and the radius is order of magnitude larger, which should lead to a decrease of the n value toward 2. Although substantial care was taken in the simulations, each run was initialized with an independent random configuration. Given the potentially very slow ion rearrangements, full equilibrium may not have been reached, and residual hydrodynamic effects could therefore still influence the observed scaling.

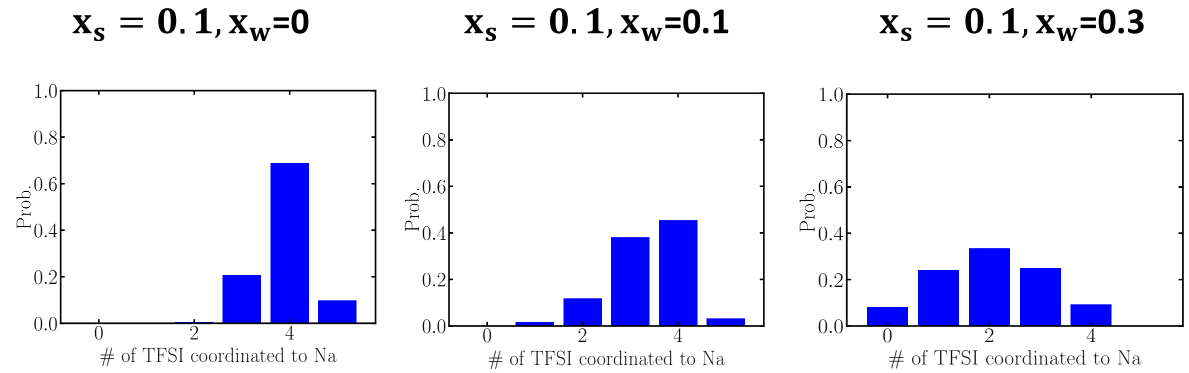

Fig. S1. Probability of different numbers of TFSI$^-$ anions coordinated to Na$^+$ cations, for the studied electrolyte systems. The three plots show that 0-5 TFSI$^-$ anions are bound to each Na$^+$ cation. For the dry SiIL, the most probable environment is 4 TFSI$^-$ anions bound to each Na$^+$ cation. This solvation environment dominates, with only a few Na$^+$ cations being bound to 3 or 5 TFSI$^-$ anions, with 0 probability of other clusters. Increasing the water content to $x_w = 0.1$ leads to 3 and 4 TFSI$^-$ anions bound to Na$^+$ with similar probabilities, and there is now a non-zero probability of associations between 1 Na$^+$ and 1 TFSI$^-$. A further increase to $x_w = 0.3$ further decreases the number of TFSI$^-$ anions bound to each Na$^+$, with a maximum probability for two anions. Note that clusters can contain more than one Na$^+$ cation, but this represents the number of TFSI$^-$ coordinated to each Na$^+$.

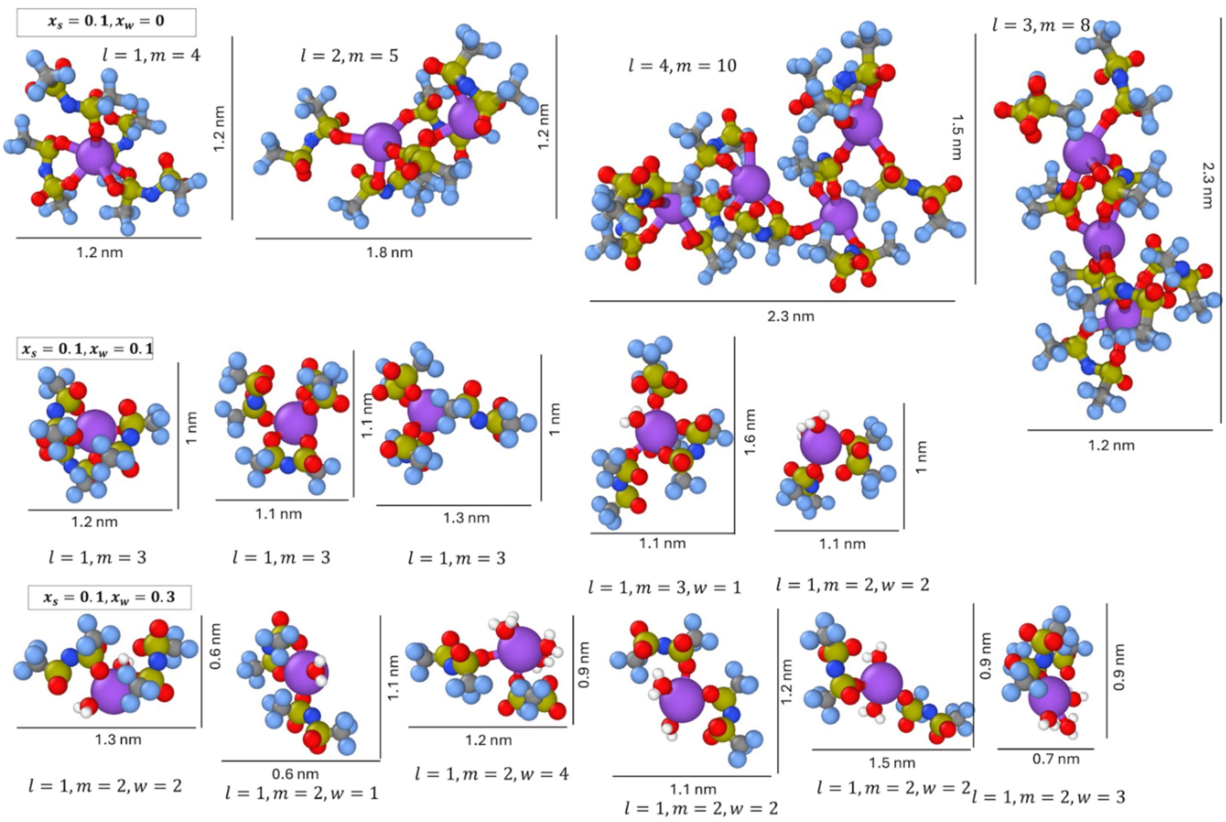

Fig. S2. Schematics of most common clusters in dry SiILs with x_s=0.1, and in water-in-SiILs with x_w= 0.1 and 0.3. The length scale of the aggregates in the dry SiILs varies from 12 to 23 Å, depending on the number of Na^+ and $TFSI^-$ ions in the aggregates. The size reported in the Main Text was roughly estimated as the geometric average of the cluster dimensions.

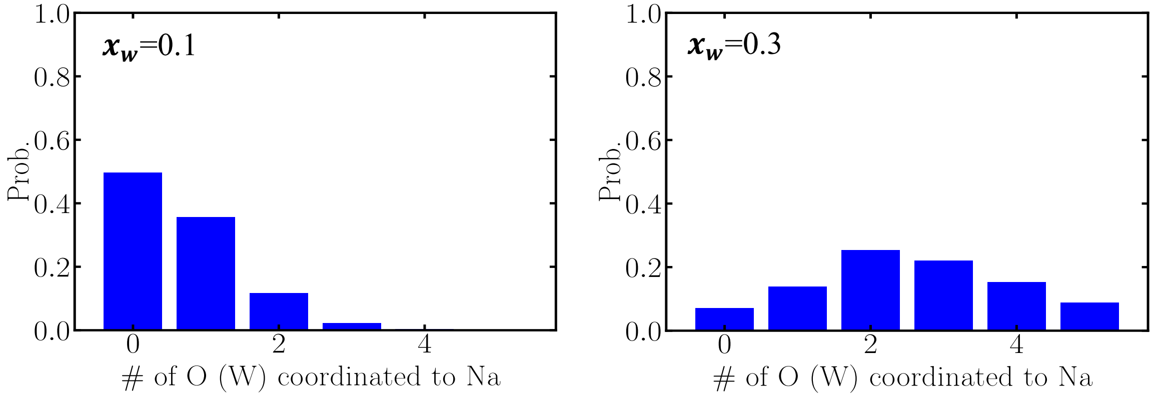

Fig. S3. Probability of water being bound to each Na^+ cation in water-in-SiILs with x_w=0.1 and x_w=0.3. For $x_w = 0.1$, approximately 50% of the Na^+ cations are not associated with water, with the remaining 50% are associated to 1-3 water molecules. At this composition, about 60% of the water molecules are not bound to any Na^+ cation. For $x_w = 0.3$, the fraction of Na^+ cations not associated with water decreases to less than 10%. Each Na^+ cation is coordinated to 1–4 water molecules, predominantly 2–3. Despite this increased coordination, approximately 65% of the water molecules remain unbound to any Na^+ cation.

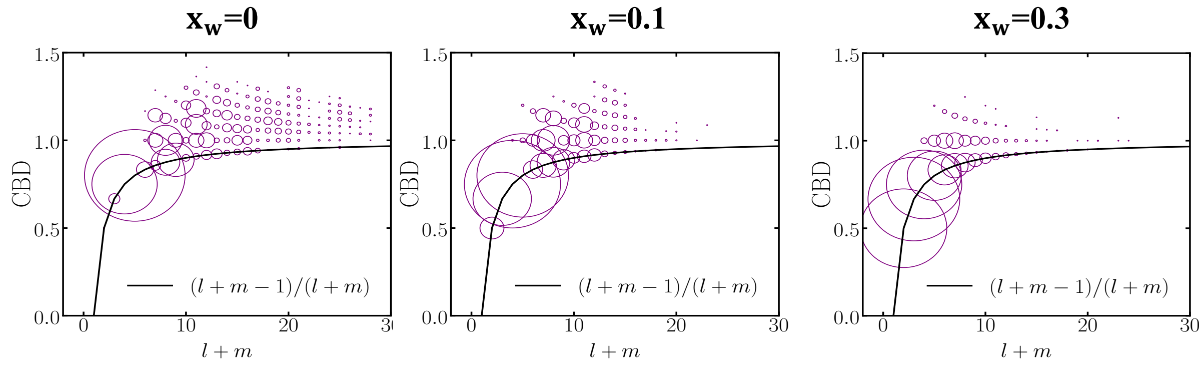

Fig. S4. Cluster bond density (CBD) as a function of the number of ions in an aggregate, $\boldsymbol{l+m}$, for the SiILs studied ($\boldsymbol{x_s}$ =0.1). The CBD is the number of bonds in a cluster divided by the number of ions in a cluster. In the ideal Cayley tree limit (shown by the black line), the CBD is equal to $(l+m-1)/(l+m)$. This is the minimum value that the CBD can take for an aggregate to exist with connected $l+m$ ions; see example in **Figure S5 (left).** Values of CBD larger than the Cayley tree limit indicate the presence of loops in the aggregate, *i.e.*, closed paths that form through additional ionic associations in the aggregates; see examples in **Figure S5 (middle, right)**. This indicates that more ordered ionic aggregates are present. At the Cayley tree limit, there are no loops present, only branched aggregates; **Figure S5 (left).** The size of each circle – for a given CBD and $l+m$ – is proportional to the number of aggregates with that specific number of ions and bonds. Therefore, larger circles denote more common aggregates. In the three electrolytes, the most likely aggregate compositions reside on the Cayley tree limit and correspond to 1 Na^+ cation with 3-5 $TFSI^-$ anions. As the size of the aggregates increases, the aggregates generally deviate from the Cayley tree limit, indicating the onset of looped (ordered) aggregates. For the dry SiIL, we showed an example of a nano-particle-like aggregate with loops in **Figure 1B**. As the water content increases, there are less larger aggregates, and a general trend toward more Cayley-tree-like aggregates. Therefore, with more water, the aggregates are becoming more disordered.

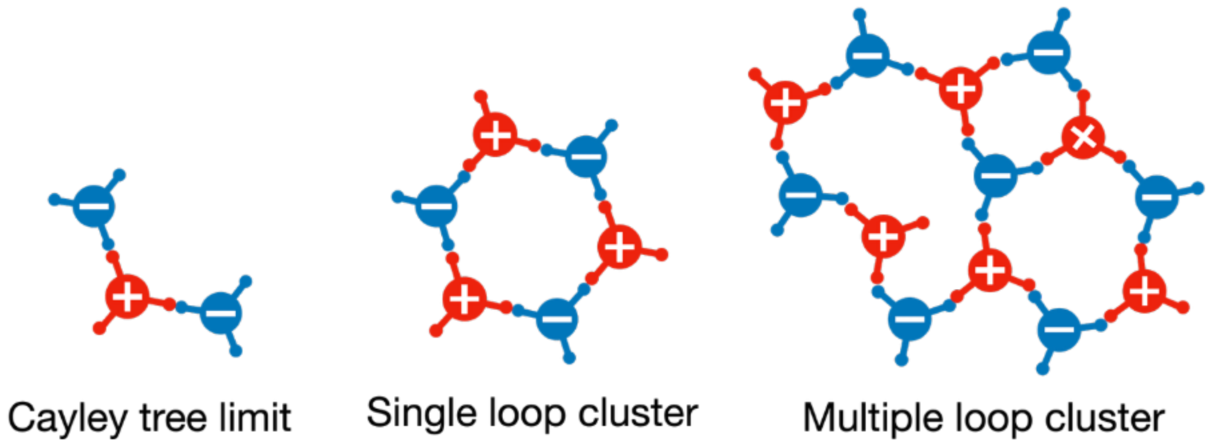

Fig. S5. Schematic representing the absence and presence of loops in clusters. In the disordered Cayley tree limit, where there is only the necessary number of associations to connect all ions in that cluster, the CBD = (l + m - 1)/(l + m), which tends to 1 for large clusters. Larger values of the CBD than the Cayley tree limit, therefore, indicate the onset of more ordered aggregates, first through the formation of loops in the clusters (closed paths of associations between ions), and then the onset of more crystalline phases were it would be more appropriate to define the aggregate in terms of a crystal packing. (Left) Example ionic aggregate in the Cayley tree limit, where the minimum number of associations connect the ions and the aggregates have a branched structure. The CBD of this aggregate is 2/3. (Middle) Example cluster with a single loop of ionic associations. The CBD of this aggregate is 1. (Right) Example cluster with multiple loops present, indicative of a more ordered ionic aggregate, above the Cayley tree limit, with a CBD equal to 14/13.

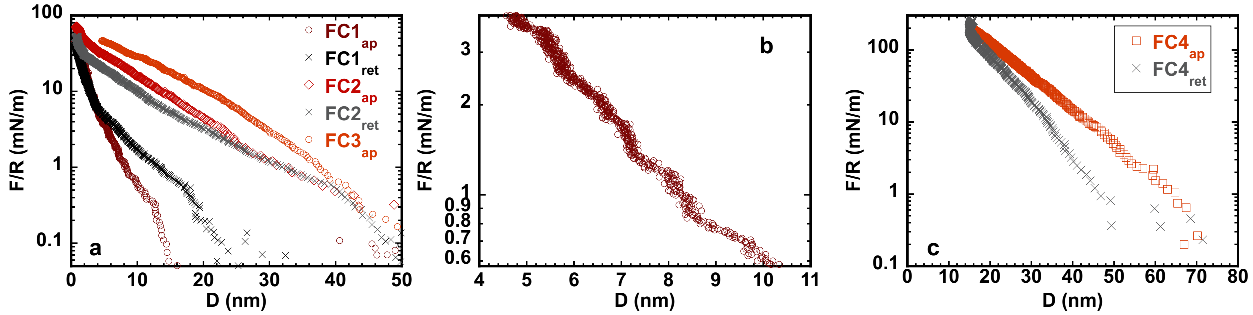

Fig. S6. Surface forces measured by SFA for the dry SiIL with other pairs of mica surfaces at 0.15 nm/s. (a) The decay length of FC1 increases in subsequent force-distance curves, leading qualitatively to similar results as in **Figure 2B**. (b) $FC1_{ap}$ also shows steps, similar to **Figure 2C**. (c) Maintaining the surfaces at a separation of D>10 µm for 24 hours and then resuming the force measurements did not lead to the recovery of $FC1_{ap}$, suggesting an irreversible transition of the electrolyte on the mica surfaces within the experimental timeframe. The differences between the force-distance profiles (FC2, and subsequent) point at slow dynamics in the SiILs (out of equilibrium), possibly transitioning gradually toward equilibrium. We hypothesize that it originates from the surface and confinement-promoted aggregation which leads to clusters with a larger characteristic length, whose compression leads to a long-range force of both steric and electrostatic nature, as well as dynamic effects. See reference (*18*) for more details.

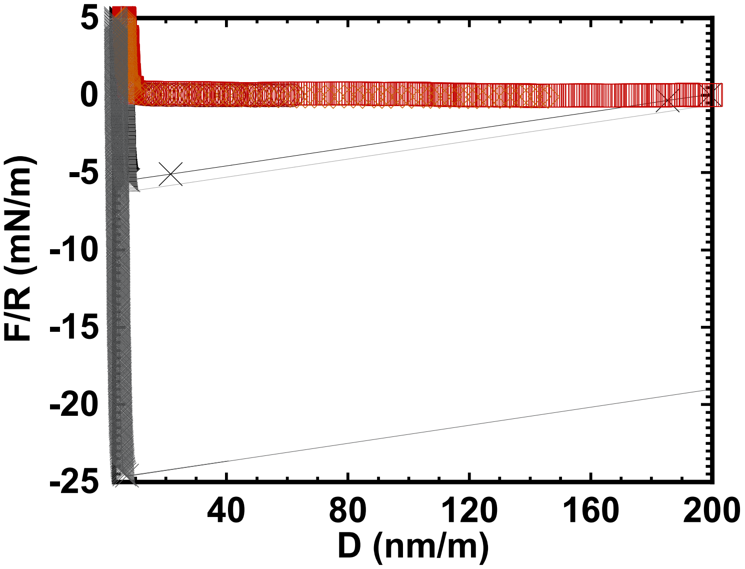

Fig. S7. Representative pull-off force between mica surfaces for water-in-SiILs with x_w=0.03 at 25ºC. Two adhesive minima are shown here, one at -5 mN/m and a second one at -25 mN/m. **Figure 2E** only shows one of them.

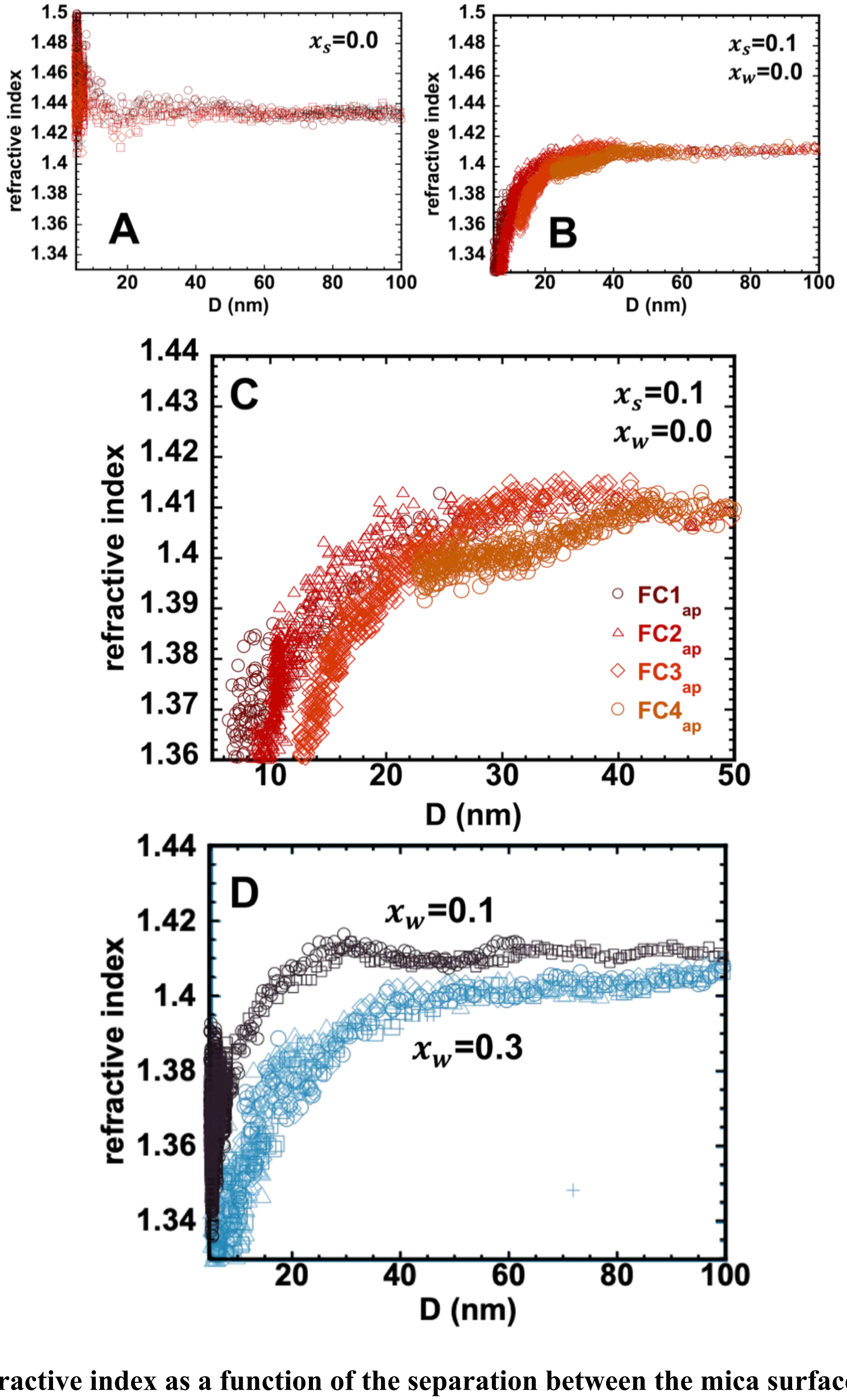

Fig. S8. Refractive index as a function of the separation between the mica surfaces (*D*) of (A) [EMIM][TFSI], (B-C) the dry SiIL with x_s=0.1 and (*D*) the water-in-SiILs with x_w=0.1 and 0.3. The refractive index of the IL determined at surface separations $D \gtrsim 60$ nm is in quantitative agreement with reported values for the bulk (unconfined) IL. Changes in refractive index at smaller surface separations are attributed to confinement effects.

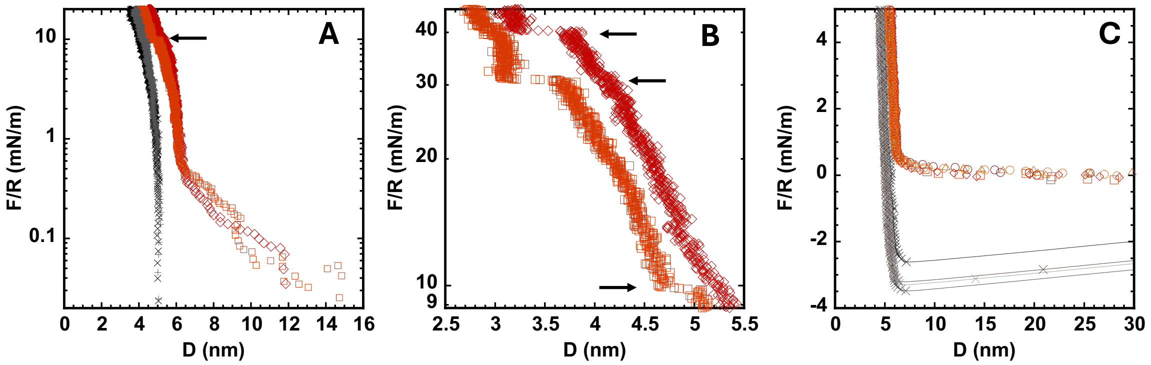

Fig. S9. Force-distance curves measured for a dry SiIL composed of x_s=0.2 NaFSI in [EMIM][FSI]. Sample preparation and SFA measurements were carried out under the same conditions as for the dry SiIL composed of x_s=0.1 NaTFSI in [EMIM][TFSI]. This means that the SFA cell is equilibrated at less than 3% RH. (A) Representative surface forces upon approach (red) and separation (black). There are some instabilities at large separations, but the decay is approximately exponential. The solubility of NaFSI in [EMIM][FSI] (x_s>0.4) is much higher than that of NaTFSI in [EMIM][TFSI]. Hence, we cannot exclude that higher concentration of NaFSI –closer to saturation– can lead to a different behavior. (B) There are layers with an average size of ~5 Å at separations smaller than 5 nm. (C) Pull-off force shown in the surface force upon retraction. The trace amounts of water in the SiIL were determined in equilibrium with dry nitrogen (214 ppm), and at 11 and 33% RH (6751 and 17643 ppm, respectively). In the SFA, the relative humidity increased up to 3% RH. The maximum water uptake by this SiIL at 3% RH was estimated by interpolation (~1627 ppm), and it is very similar to that of the dry TFSI-SiIL at x_s=0.1. Consequently, the different surface forces cannot be attributed to the water content. The viscosity of the SiIL is 36 mPa.s, which is smaller than that of x_s=0.1 NaTFSI in [EMIM][TFSI] (59.5 mPa.s from ref.(*18*)). As described later, this should lead to 2× higher hydrodynamic drag.

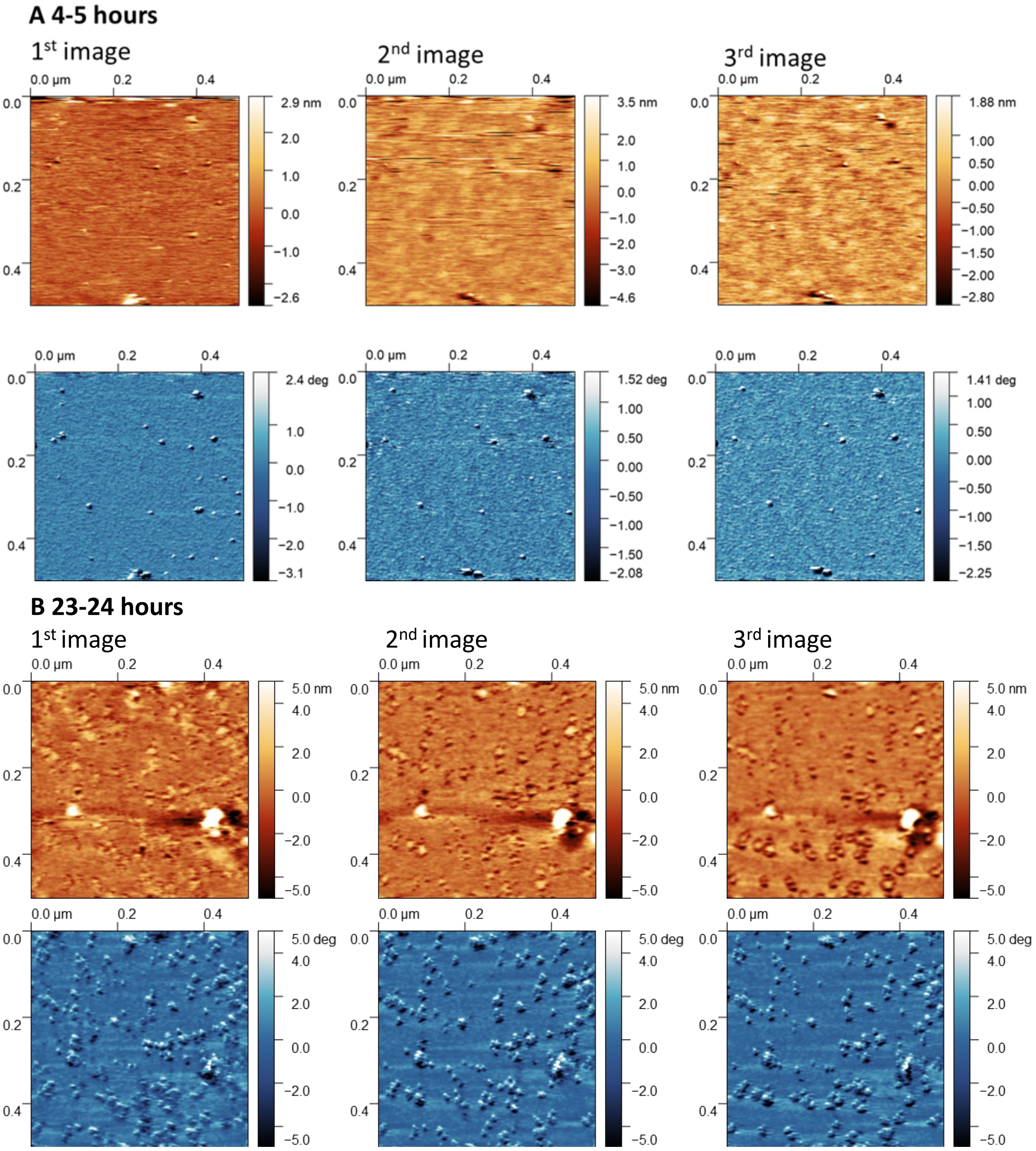

Fig. S10. Tapping mode AFM images of topography (red) and phase (blue) of mica immersed in the dry SiIL with x_s= 0.10 NaTFSI after 4-5 hours (A) and 23-24 hours (B). Three consecutive scans (phase) on the same spot to illustrate that the aggregates remain on the surface.

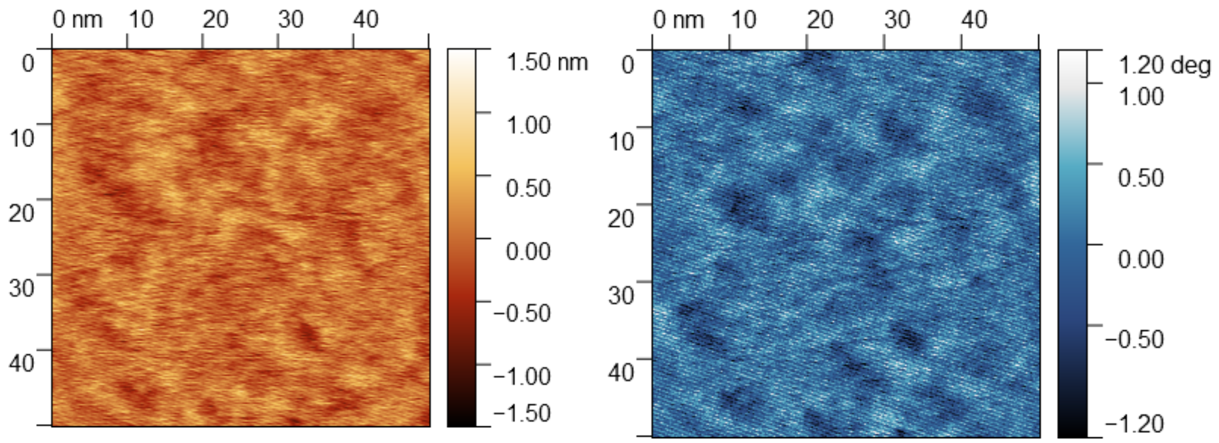

Fig. S11. Tapping mode AFM images of topography (red) and phase (blue) of mica immersed in a water-in-SiIL with x_s= 0.10 NaTFSI equilibrated at 33% RH (x_w = 0.3) at room temperature. Higher magnifications images than in **Figure 3** after equilibration for 23-24 hours to confirm the absence of aggregates.

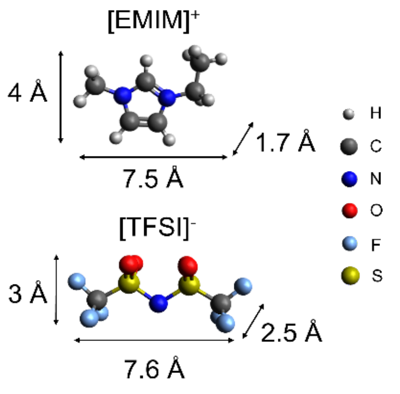

Fig. S12. Molecular structure of $[EMIM]^+$ and $[TFSI]^-$. The dimensions of the ions were determined by the software Avogadro 1.2.0 using MMFF94 as the force field for energy minimization.

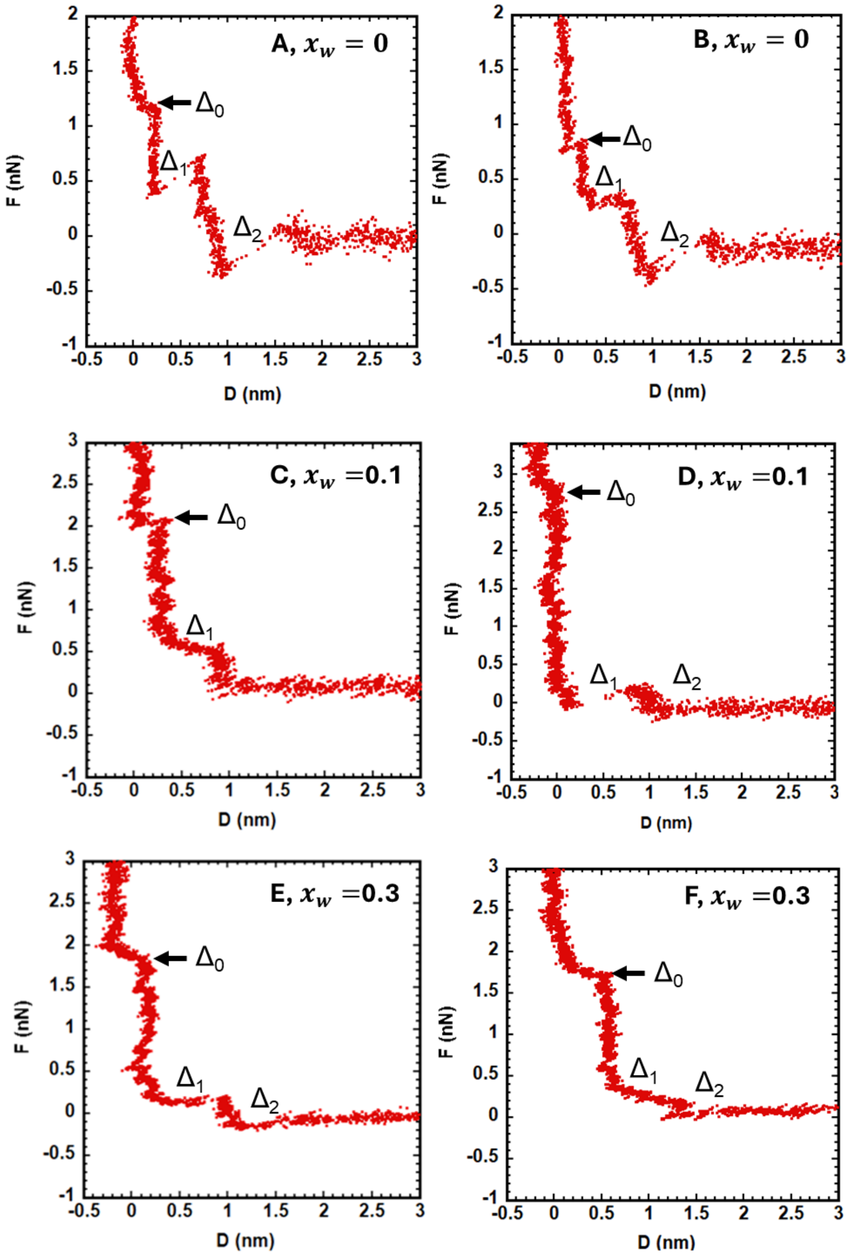

Fig. S13. Single force-distance curves measured by AFM showing a step (Δ_0) at high force in SiILs with x_s = 0.10 NaTFSI and water contents (A-B) x_w=0, (C-D) x_w=0.1, and (E-F) x_w=0.3.

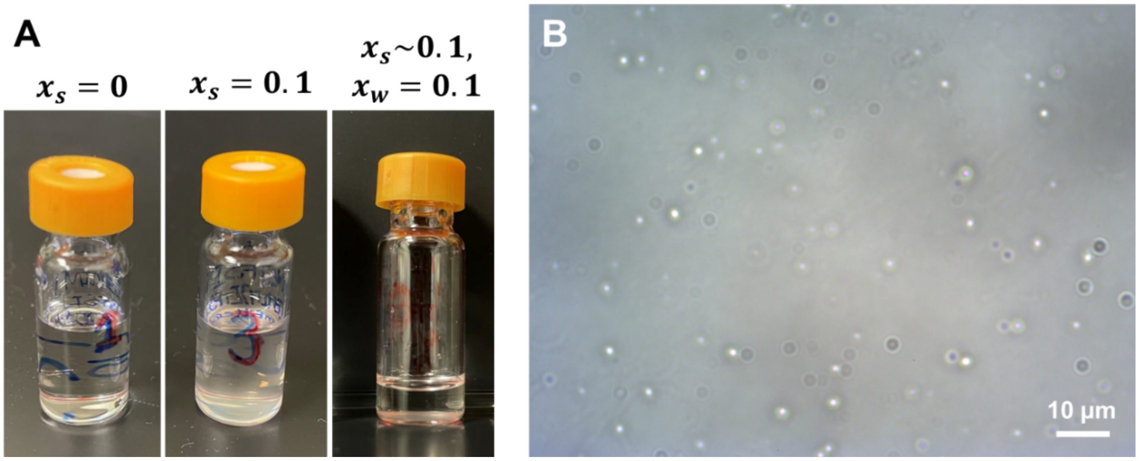

Fig. S14. Liquid-liquid phase separation ((A) Pictures of dry [EMIM][TFSI] (x_s=0), x_s=0.1 SiIL, and x_s~0.1, x_w=0.1 water-in-SiIL, and (B) 50× magnification optical image of the turbid dry SiIL with x_s=0.1 before filtering. The dry SiILs were turbid due to the presence of micrometer-sized droplets. The volume percentage of droplets was estimated by counting the number of droplets per image area and is <0.8 vol%, as dynamic light scattering was not able to detect them. The droplets are not birefringent and were filtered prior to the experimental studies.

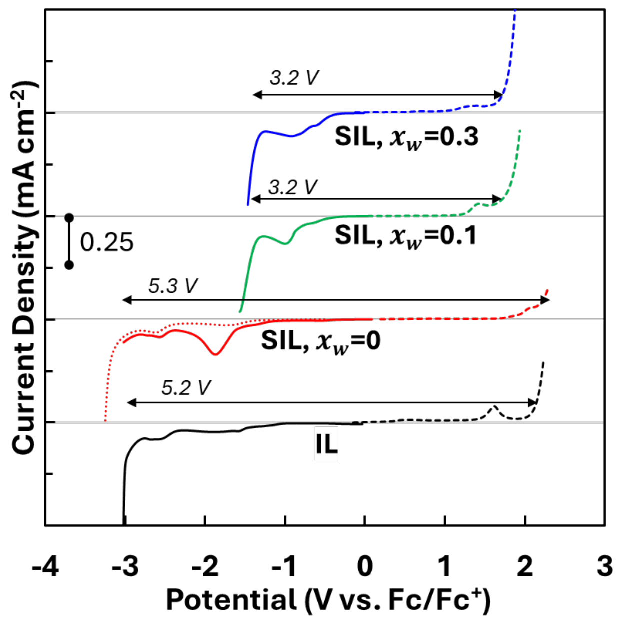

Fig. S15. Linear sweep voltammograms (LSVs) measured on a gold working electrode (WE). Both scans (i.e. toward positive and negative potentials) start from the open circuit potential on pristine gold WE. Solid lines correspond to the first sweeps toward negative potentials; the dotted line corresponds to the second sweep toward negative limit; dashed lines correspond to the first sweeps toward positive potentials. Scan rate: 10 mV s^{-1}.

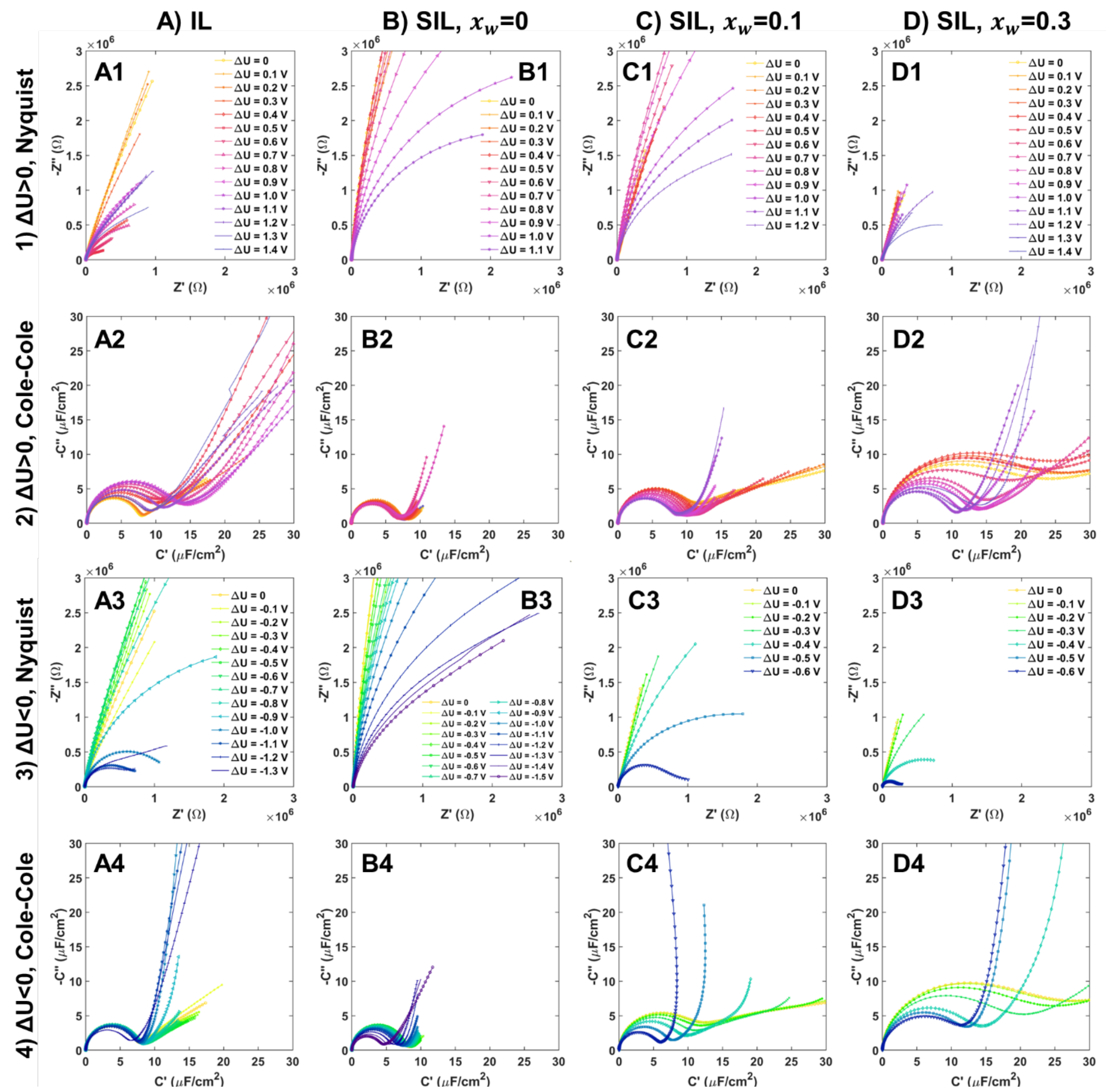

Fig. S16. **Nyquist and Cole-Cole plots** for (A) IL, (B) dry SiIL, (C) SiIL with x_w=0.1, and (D) x_w=0.3 with 1) ΔU (potential respect to OCP) >0, Nyquist, 2) ΔU>0, Cole-Cole, 3) ΔU<0, Nyquist, and 4) ΔU<0, Cole-Cole plots. The semicircle in the Cole-Cole plot indicates the double layer charging process and is the targeted frequency range for fitting to obtain double layer capacitance.

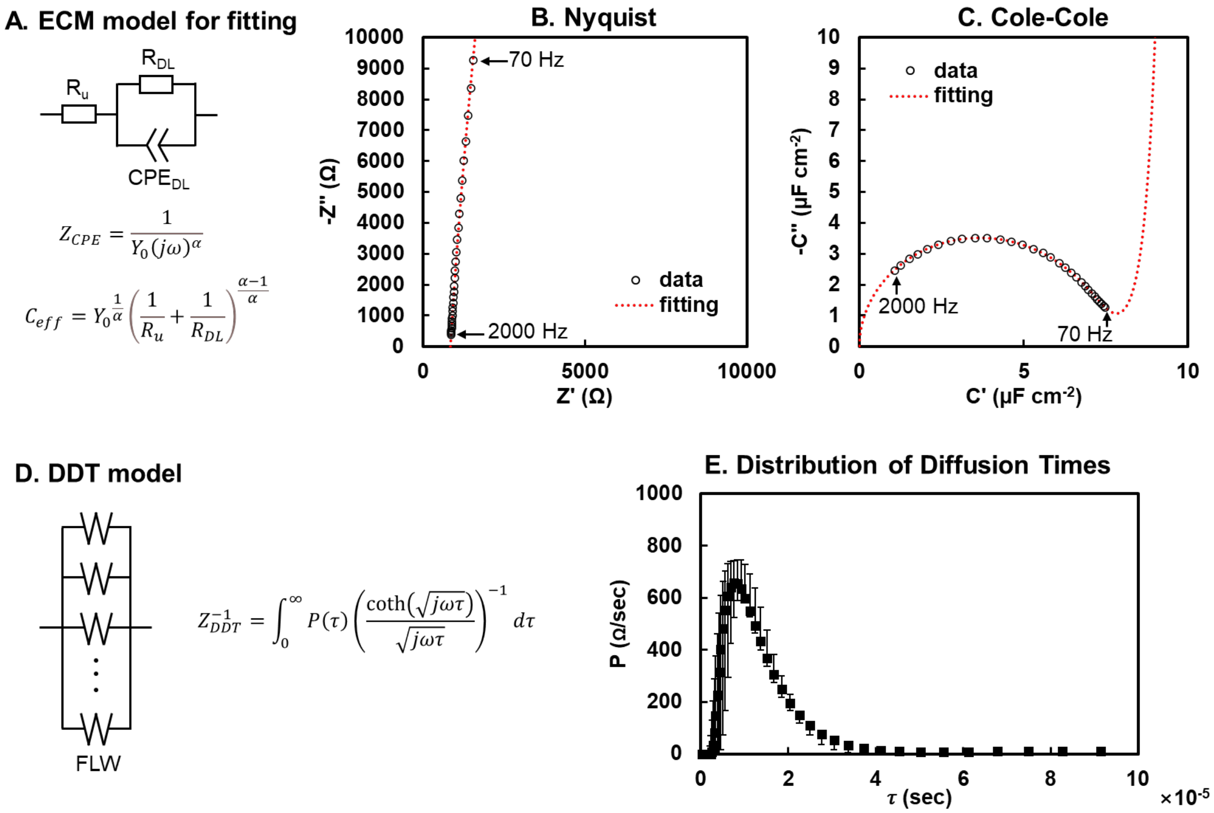

Fig. S17. Electric models applied to determine the double layer capacitance. (A) Electric circuit model used to fit the data within the double layer charging frequency range shown in **Figure S16**. Here, R_u represents the resistance of the bulk electrolyte, R_{DL} is the resistance of the double layer, and CPE_{DL} describes a non-ideal double layer. The example data shown in (B) and (C) is for the dry IL at open circuit potential. The effective capacitance associated with the CPE is calculated using the method first developed by Brug et al.(*77*) and explained by Hirschorn et al.(*78*) in more detail. The CPE behavior could hypothetically be attributed to a distribution of diffusion times (DDT) through the double layer, arising from diffusive rearrangements of the ionic clusters. Preliminary results of applying the DDT model,(*79*) illustrated in (D), are presented in (E) as a distribution of diffusion times estimated from the same dataset. We are further examining this hypothesis with extended datasets in a separate, dedicated study.

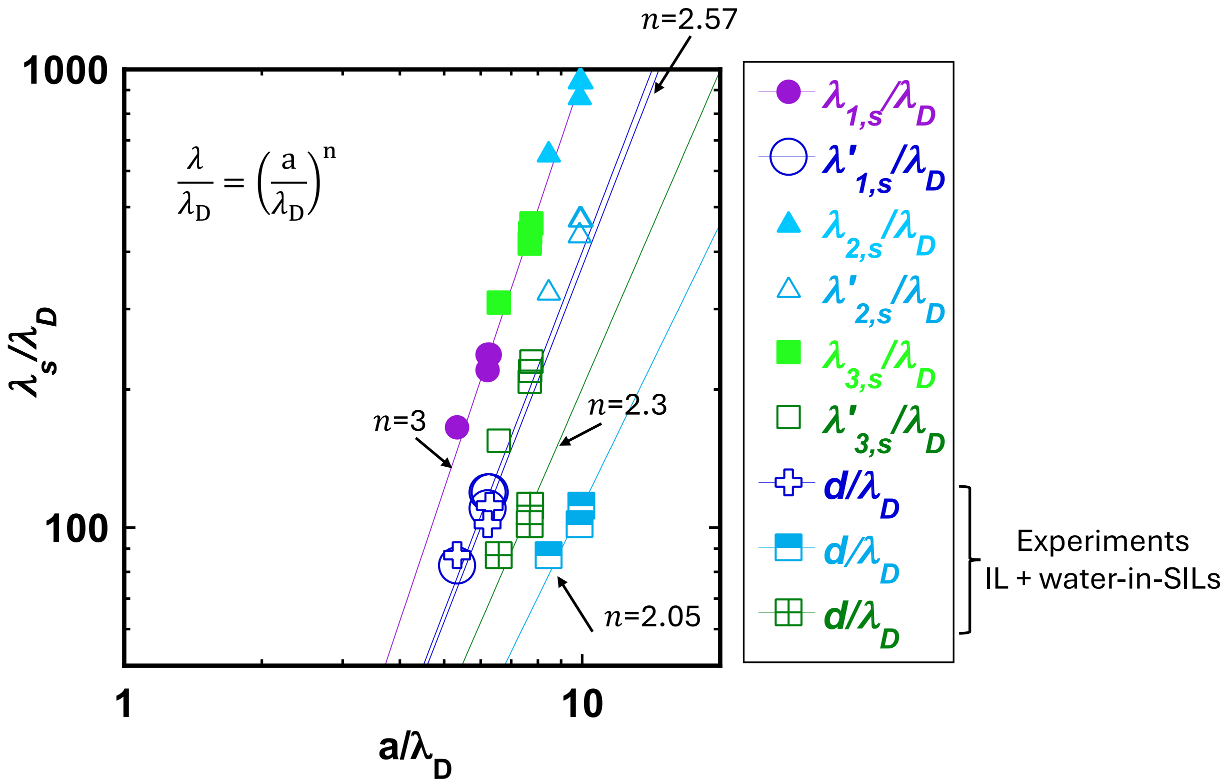

Fig. S18. Comparison between experimental decay length and calculation length using the anomalous underscreening model. We compare here methods 1-3 to determine the mean ion diameter: method 1 in dark blue, method 2 in light blue and method 3 in green. The full markers correspond to λ_s and the empty markers to λ_s'. The experimental results are shown for the decay length of the surface force [EMIM][]TFSI] (d) and the two water-in-SiILs vs. $\frac{a_1}{\lambda_D}$, $\frac{a_2}{\lambda_D}$ and $\frac{a_3}{\lambda_D}$. The values for the dry SiIL are not shown due to very strong dynamic effects.

sample	A_2 (-)	q_2 (Å^{-1})	error	l_2 (Å)	error	B_2 (Å^{-1})	B_2 (Å^{-1}) *corrected*	error	A_3	q_3 (Å^{-1})	error	l_3 (Å)	error	B_3 (Å^{-1})	B_3 (Å^{-1}) *corrected*	error	R^2
x_s=0	0.20992	0.89336	0.00058	7.03321	0.00453	0.12800	0.075	0.00125	0.28287	1.36150	0.00121	4.61490	0.00410	0.38180	0.329	0.00282	0.99932
x_s=0.1	0.15556	0.88468	0.00081	7.10221	0.00648	0.17421	0.121	0.00179	0.27128	1.35270	0.00124	4.64492	0.00424	0.38994	0.337	0.00241	0.99955
x_s=0.1 x_w=0.1	0.11718	0.89632	0.00070	7.00998	0.00547	0.16145	0.108	0.00157	0.19204	1.36260	0.00122	4.61117	0.00413	0.39598	0.343	0.00251	0.99956
x_s=0.1 x_w=0.3	0.164	0.90278	0.00064	6.95982	0.00493	0.14028	0.087	0.00147	0.23032	1.36710	0.00147	4.59600	0.00496	0.41192	0.359	0.00340	0.99936

Table S1. Fitting parameters of the Lorentzian functions with two peaks to x-ray scattering intensity. The parameters are q_i in Å^{-1}, and B_i in Å^{-1}, with the corresponding errors in the same units. The d-spacing was determined from $l_i = 2\pi/q_i$ and the error was determined from error propagation; both are given in Å. B_i is the half-width at half-maximum, which was corrected with the instrumental broadening for the four electrolytes.

x_s/x_w	peak #	μ [nm]	σ [nm]	contribution
x_s = 0 NaTFSI<br>x_w = 0	Gaussian 1	-0.00036	0.07302	0.36300
	Gaussian 2	0.49784	0.09520	0.31142
	Gaussian 3	1.24303	0.12043	0.12400
	Gaussian 4	1.98637	0.14804	0.07382
	Gaussian 5	2.71612	0.19092	0.05968
	Gaussian 6	3.50147	0.27350	0.06809
x_s= 0.1 NaTFSI<br>x_w = 0	Gaussian 1	0.07651	0.06346	0.46821
	Gaussian 2	0.88328	0.13743	0.27779
	Gaussian 3	1.82259	0.21573	0.15471
x_s= 0.1 NaTFSI<br>x_w = 0.1	Gaussian 1	0.05320	0.11522	0.38398
	Gaussian 2	0.52681	0.21153	0.24089
	Gaussian 3	1.56249	0.43098	0.14971
	Gaussian 4	2.80269	0.47311	0.16267
	Gaussian 5	3.68502	0.20271	0.06275
x_s= 0.1 NaTFSI<br>x_w = 0.3	Gaussian 1	0.18916	0.16504	0.31799
	Gaussian 2	0.96466	0.27002	0.33661
	Gaussian 3	2.07464	0.51097	0.19361
	Gaussian 4	3.12943	0.35693	0.10773
	Gaussian 5	3.78019	0.14059	0.04406

Table S2. Parameters of a Gaussian mixture distribution model fitted to the 1-D histograms of AFM data. For each single Gaussian distribution, the mean value (μ), the standard deviation (σ), and the relative contribution were obtained. All the fits lead to R^2 values larger than 99%.

					method 1			method 2			method 3			
	**ion pair concentration (mol/m^3)**	**density (g/cm^3)**	**molecular volume (Å^3)**	λ_D **(nm)**	a_1 (Å)	$\lambda_{s,1}$ **(nm)**	$\lambda_{s,1}{}'$ **(nm)**	a_2 (Å)	$\lambda_{s,2}$ **(nm)**	$\lambda_{s,2}{}'$ **(nm**	a_3 (Å)	$\lambda_{s,3}$ **(nm)**	$\lambda_{s,3}{}'$ **(nm)**	d **(nm)**
**[EMIM][TFSI]**	3884.4	1.520	427.43	0.06	3.77	14.32	7.16	5.98	57.29	28.64	4.62	26.35	13.17	6.7 +/-1.0
x_s**=0.1**	4000.1	1.530	415.07	0.06	3.73	14.32	7.16	5.92	57.29	28.64	4.65	27.66	13.83	–
x_s**=0.1** x_w**=0.1**	3843.1	1.477	432.02	0.06	3.73	13.22	6.61	5.92	52.89	26.44	4.61	24.98	12.49	6.12 $\pm$ 1.2
x_s**=0.1** x_w**=0.3**	3752.4	1.460	442.46	0.07	3.73	11.57	5.79	5.92	46.30	23.15	4.60	21.66	10.83	6.18 $\pm$ 0.9

Table S3. Scaling analysis of anomalous underscreening. See details in SI Text.

REFERENCES

1. K. Xu, Nonaqueous liquid electrolytes for lithium-based rechargeable batteries. *Chem. Rev.* **104**, 4303–4418 (2004).

2. K. Xu, Electrolytes and interphases in Li-ion batteries and beyond. *Chem. Rev.* **114**, 11503–11618 (2014).

3. T. Welton, Room-temperature ionic liquids. Solvents for synthesis and catalysis. *Chem. Rev.* **99**, 2071–2084 (1999).

4. G. G. Eshetu, G. A. Elia, M. Armand, M. Forsyth, S. Komaba, T. Rojo, S. Passerini, Electrolytes and interphases in sodium-based rechargeable batteries: Recent advances and perspectives. *Adv. Energy Mater.* **10**, 2000093 (2020).

5. D. Monti, E. Jónsson, M. R. Palacín, P. Johansson, Ionic liquid based electrolytes for sodium-ion batteries: Na^+ solvation and ionic conductivity. *J. Power Sources* **245**, 630–636 (2014).

6. G. A. Giffin, A. Moretti, S. Jeong, S. Passerini, Complex nature of ionic coordination in magnesium ionic liquid-based electrolytes: Solvates with mobile Mg^{2+} cations. *J. Phys. Chem. C* **118**, 9966–9973 (2014).

7. Z. Liu, G. Pulletikurthi, A. Lahiri, T. Cui, F. Endres, Suppressing the dendritic growth of zinc in an ionic liquid containing cationic and anionic zinc complexes for battery applications. *Dalton Trans.* **45**, 8089–8098 (2016).

8. M. Gouverneur, F. Schmidt, M. Schonhoff, Negative effective Li transference numbers in Li salt/ionic liquid mixtures: Does Li drift in the “Wrong” direction? *Phys. Chem. Chem. Phys.* **20**, 7470–7478 (2018).

9. N. Molinari, J. P. Mailoa, B. Kozinsky, General trend of a negative Li effective charge in ionic liquid electrolytes. *J. Phys. Chem. Lett.* **10**, 2313–2319 (2019).

10. P. Kubisiak, P. Wrobel, A. Eilmes, Molecular dynamics investigation of correlations in ion transport in MeTFSI/EMIM-TFSI (Me = Li, Na) electrolytes. *J. Phys. Chem. B* **124**, 413–421 (2020).

11. M. McEldrew, Z. A. H. Goodwin, N. Molinari, B. Kozinsky, A. A. Kornyshev, M. Z. Bazant, Salt-in-ionic-liquid electrolytes: ion network formation and negative effective charges of alkali metal cations. *J. Phys. Chem. B* **125**, 13752–13766 (2021).

12. M. McEldrew, Z. A. H. Goodwin, S. Bi, A. A. Kornyshev, M. Z. Bazant, Ion clusters and networks in water-in-salt electrolytes. *J. Electrochem. Soc.* **168**, 050514 (2021).

13. D. M. Pesko, K. Timachova, R. Bhattacharya, M. C. Smith, I. Villaluenga, J. Newman, N. P. Balsara, Negative transference numbers in poly(ethylene oxide)-based electrolytes. *J. Electrochem. Soc.* **164**, E3569–E3575 (2017).

14. K. Shigenobu, M. Shibata, K. Dokko, M. Watanabe, K. Fujii, K. Ueno, Anion effects on Li ion transference number and dynamic ion correlations in glyme-Li salt equimolar mixtures. *Phys. Chem. Chem. Phys.* **23**, 2622–2629 (2021).

15. Y. Shao, H. Gudla, D. Brandell, C. Zhang, Transference number in polymer electrolytes: Mind the reference-frame gap. *J. Am. Chem. Soc.* **144**, 7583–7587 (2022).

16. J. Im, C. Fang, D. M. Halat, S. Chakraborty, D. T. Hickson, I. Woolsey, Q. N. Dao, R. Wang, J. A. Reimer, N. P. Balsara, Solvation governs cation transference in glyme-based lithium battery electrolytes. *J. Chem. Phys.* **162**, 214705 (2025).

17. R. M. Espinosa-Marzal, Z. A. H. Goodwin, X. Zhang, Q. Zheng, in *One Hundred Years of Colloid Symposia: Looking Back and Looking Forward*. (American Chemical Society, 2023), vol. 1457, chap. 7, pp. 123–148.

18. X. Zhang, Z. A. H. Goodwin, A. G. Hoane, A. Deptula, D. M. Markiewitz, N. Molinari, Q. Zheng, H. Li, M. McEldrew, B. Kozinsky, M. Z. Bazant, C. Leal, R. Atkin, A. A. Gewirth, M. W. Rutland, R. M. Espinosa-Marzal, Long-range surface forces in salt-in-ionic liquids. *ACS Nano* **18**, 34007–34022 (2024).

19. M. A. Gebbie, M. Valtiner, X. Banquy, E. T. Fox, W. A. Henderson, J. N. Israelachvili, Ionic liquids behave as dilute electrolyte solutions. *Proc. Natl. Acad. Sci. U.S.A.* **110**, 9674–9679 (2013).

20. R. M. Espinosa-Marzal, A. Arcifa, A. Rossi, N. D. Spencer, Microslips to "Avalanches" in confined, molecular layers of ionic liquids. *J. Phys. Chem. Lett.* **5**, 179–184 (2014).

21. A. A. Lee, C. S. Perez-Martinez, A. M. Smith, S. Perkin, Scaling analysis of the screening length in concentrated electrolytes. *Phys. Rev. Lett.* **119**, 026002 (2017).

22. J. G. Kirkwood, Statistical mechanics of liquid solutions. *Chem. Rev.* **19**, 275–307 (2002).

23. A. A. Lee, C. S. Perez-Martinez, A. M. Smith, S. Perkin, Underscreening in concentrated electrolytes. *Faraday Discuss.* **199**, 239–259 (2017).

24. B. Cross, L. Garcia, E. Charlaix, P. Kekicheff, Short-range electrostatic screening in ionic liquids as inferred by direct force measurements. *Proc. Natl. Acad. Sci. U.S.A.* **123**, e2517939123 (2026).

25. W. W. Wang, Y. Gu, H. Yan, K. X. Li, Z. B. Chen, Q. H. Wu, C. Kranz, J. W. Yan, B. W. Mao, Formation sequence of solid electrolyte interphases and impacts on lithium deposition and dissolution on copper: An in situ atomic force microscopic study. *Faraday Discuss.* **233**, 190–205 (2022).

26. S. A. Ferdousi, L. A. O'Dell, M. Hilder, A. J. Barlow, M. Armand, M. Forsyth, P. C. Howlett, SEI formation on sodium metal electrodes in superconcentrated ionic liquid electrolytes and the effect of additive water. *ACS Appl. Mater. Interfaces* **13**, 5706–5720 (2021).

27. D. R. MacFarlane, P. Meakin, J. Sun, N. Amini, M. Forsyth, Pyrrolidinium imides: A new family of molten salts and conductive plastic crystal phases. *J. Phys. Chem. B* **103**, 4164–4170 (1999).

28. P. Kubisiak, A. Eilmes, Molecular dynamics simulations of ionic liquid based electrolytes for na-ion batteries: Effects of force field. *J. Phys. Chem. B* **121**, 9957–9968 (2017).

29. L. Hakim, Y. Ishii, K. Matsumoto, R. Hagiwara, K. Ohara, Y. Umebayashi, N. Matubayasi, Transport properties of ionic liquid and sodium salt mixtures for sodium-ion battery electrolytes from molecular dynamics simulation with a self-consistent atomic charge determination. *J. Phys. Chem. B* **124**, 7291–7305 (2020).

30. S. Kunigal Vijaya Shankar, Y. Claveau, T. Rasoanarivo, C. Ewels, J. Le Bideau, Impact of Li, Na and Zn metal cation concentration in EMIM–TFSI ionic liquids on ion clustering, structure and dynamics. *Phys. Chem. Chem. Phys.* **26**, 7049–7059 (2024).

31. D. Reber, R. Figi, R.-S. Kühnel, C. Battaglia, Stability of aqueous electrolytes based on LiFSI and NaFSI. *Electrochim. Acta* **321**, 134644 (2019).

32. S. F. Lux, L. Terborg, O. Hachmöller, T. Placke, H. W. Meyer, S. Passerini, M. Winter, S. Nowak, LiTFSI stability in water and its possible use in aqueous lithium-ion batteries: pH dependency, electrochemical window and temperature stability. *J. Electrochem. Soc.* **160**, A1694–A1700 (2013).

33. D. M. Markiewitz, Z. A. H. Goodwin, Q. Zheng, M. McEldrew, R. M. Espinosa-Marzal, M. Z. Bazant, Ionic associations and hydration in the electrical double layer of water-in-salt electrolytes. *ACS Appl. Mater. Interfaces* **17**, 29515–29534 (2025).

34. J. J. Hettige, J. C. Araque, C. J. Margulis, Bicontinuity and multiple length scale ordering in triphilic hydrogen-bonding ionic liquids. *J. Phys. Chem. B* **118**, 12706–12716 (2014).

35. O. Russina, A. Triolo, L. Gontrani, R. Caminiti, D. Xiao, L. G. Hines Jr, R. A. Bartsch, E. L. Quitevis, N. Pleckhova, K. R. Seddon, Morphology and intermolecular dynamics of 1-alkyl-3-methylimidazolium bis{(trifluoromethane)sulfonyl}amide ionic liquids: Structural and dynamic evidence of nanoscale segregation. *J. Phys.:Condens. Matter* **21**, 424121 (2009).

36. R. Lhermerout, S. Perkin, Nanoconfined ionic liquids: Disentangling electrostatic and viscous forces. *Phys. Rev. Fluids* **3**, 014201 (2018).

37. N. Molinari, J. P. Mailoa, N. Craig, J. Christensen, B. Kozinsky, Transport anomalies emerging from strong correlation in ionic liquid electrolytes. *J. Power Sources* **428**, 27–36 (2019).

38. A. M. Smith, K. R. Lovelock, N. N. Gosvami, T. Welton, S. Perkin, Quantized friction across ionic liquid thin films. *Phys. Chem. Chem. Phys.* **15**, 15317–15320 (2013).

39. M. Han, R. M. Espinosa-Marzal, Influence of water on structure, dynamics, and electrostatics of hydrophilic and hydrophobic ionic liquids in charged and hydrophilic confinement between mica surfaces. *ACS Appl. Mater. Interfaces* **11**, 33465–33477 (2019).

40. M. Heuberger, The extended surface forces apparatus. Part I. Fast spectral correlation interferometry. *Rev. Sci. Instrum.* **72**, 1700–1707 (2001).

41. A. P. Froba, H. Kremer, A. Leipertz, Density, refractive index, interfacial tension, and viscosity of ionic liquids [EMIM][$EtSO_4$], [EMIM][NTf_2], [EMIM][$N(CN)_2$], and [OMA][NTf_2] in dependence on temperature at atmospheric pressure. *J. Phys. Chem. B* **112**, 12420–12430 (2008).

42. U. Raviv, J. Frey, R. Sak, P. Laurat, R. Tadmor, J. Klein, Properties and interactions of physigrafted end-functionalized poly (ethylene glycol) layers. *Langmuir* **18**, 7482–7495 (2002).

43. O. Borodin, W. Gorecki, G. D. Smith, M. Armand, Molecular dynamics simulation and pulsed-field gradient NMR studies of bis(fluorosulfonyl)imide (FSI) and bis[(trifluoromethyl)sulfonyl]imide (TFSI)-based ionic liquids. *J. Phys. Chem. B* **114**, 6786–6798 (2010).

44. A. M. Smith, S. Perkin, Influence of lithium solutes on double-layer structure of ionic liquids. *J. Phys. Chem. Lett.* **6**, 4857–4861 (2015).

45. J. B. Haskins, J. J. Wu, J. W. Lawson, Computational and experimental study of Li-doped ionic liquids at electrified interfaces. *J. Phys. Chem. C. Nanomater. Interfaces* **120**, 11993–12011 (2016).

46. R. Atkin, G. G. Warr, Structure in confined room-temperature ionic liquids. *J. Phys. Chem. C* **111**, 5162–5168 (2007).

47. R. Hayes, S. Z. El Abedin, R. Atkin, Pronounced structure in confined aprotic room-temperature ionic liquids. *J. Phys. Chem. B* **113**, 7049–7052 (2009).

48. S. McDonald, A. Elbourne, G. G. Warr, R. Atkin, Metal ion adsorption at the ionic liquid-mica interface. *Nanoscale* **8**, 906–914 (2016).

49. M. Heuberger, M. Zäch, Nanofluidics: Structural forces, density anomalies, and the pivotal role of nanoparticles. *Langmuir* **19**, 1943–1947 (2003).

50. S. J. O'Shea, N. N. Gosvami, L. T. Lim, W. Hofbauer, Liquid atomic force microscopy: Solvation forces, molecular order, and squeeze-out. *Jpn. J. Appl. Phys.* **49**, 08LA01 (2010).

51. J. M. Klein, E. Panichi, B. Gurkan, Potential dependent capacitance of [EMIM][TFSI], [N(1114)][TFSI] and [PYR(13)][TFSI] ionic liquids on glassy carbon. *Phys. Chem. Chem. Phys.* **21**, 3712–3720 (2019).

52. A. M. Smith, A. A. Lee, S. Perkin, The electrostatic screening length in concentrated electrolytes increases with concentration. *J. Phys. Chem. Lett.* **7**, 2157–2163 (2016).

53. G. R. Elliott, K. P. Gregory, H. Robertson, V. S. J. Craig, G. B. Webber, E. J. Wanless, A. J. Page, The known-unknowns of anomalous underscreening in concentrated electrolytes. *Chem. Phys. Lett.* **843**, 141190 (2024).

54. E. Krucker-Velasquez, M. Z. Bazant, A. Alexander-Katz, J. W. Swan, Potential of mean force and underscreening of polarizable colloids in concentrated electrolytes. *Nano Lett.* **25**, 10362–10368 (2025).

55. J. P. de Souza, Z. A. H. Goodwin, M. McEldrew, A. A. Kornyshev, M. Z. Bazant, Interfacial layering in the electric double layer of ionic liquids. *Phys. Rev. Lett.* **125**, 116001 (2020).

56. M. Z. Bazant, L. Bocquet, F. Cicoira, D. F. Duarte Sanchez, E. Farrell, C. Holm, S. Igor, A. Janardanan, F. Jimenez-Angeles, R. Johnson, T. M. Kamsma, F. Kanoufi, A. A. Kornyshev, S. G. Lemay, Y. Levin, S. Marbach, M. Olvera de la Cruz, S. Perkin, G. Pireddu, P. Robin, B. Rotenberg, A. Schlaich, Z. S. Siwy, D. Stein, A. Thorneywork, M. Valtiner, R. van Roij, G. Yossifon, Y. Zhang, Iontronics under confinement: General discussion. *Faraday Discuss.* **246**, 592–617 (2023).

57. M. Han, R. M. Espinosa-Marzal, Electroviscous retardation of the squeeze out of nanoconfined ionic liquids. *J. Phys. Chem. C* **122**, 21344–21355 (2018).

58. H. Row, J. B. Fernandes, K. K. Mandadapu, K. Shekhar, Spatiotemporal dynamics of ionic reorganization near biological membrane interfaces. *Phys. Rev. Res.* **7**, 013185 (2025).

59. D. M. Markiewitz, Z. A. H. Goodwin, M. McEldrew, J. Pedro de Souza, X. Zhang, R. M. Espinosa-Marzal, M. Z. Bazant, Electric field induced associations in the double layer of salt-in-ionic-liquid electrolytes. *Faraday Discuss.* **253**, 365–384 (2024).

60. Z. L. Seeger, R. Kobayashi, E. I. Izgorodina, Cluster approach to the prediction of thermodynamic and transport properties of ionic liquids. *J. Chem. Phys.* **148**, 193832 (2018).

61. R. Kjellander, R. Ramirez, Yukawa multipole electrostatics and nontrivial coupling between electrostatic and dispersion interactions in electrolytes. *J. Phys. Condens. Matter* **20**, 494209 (2008).

62. D. F. Parsons, B. W. Ninham, Surface charge reversal and hydration forces explained by ionic dispersion forces and surface hydration. *Colloids Surf. A Physicochem. Eng. Asp.* **383**, 2–9 (2011).

63. D. Fedunova, A. Antosova, J. Marek, V. Vanik, E. Demjen, Z. Bednarikova, Z. Gazova, Effect of 1-ethyl-3-methylimidazolium tetrafluoroborate and acetate ionic liquids on stability and amyloid aggregation of lysozyme. *Int. J. Mol. Sci.* **23**, 783 (2022).

64. M. Han, R. Zhang, A. A. Gewirth, R. M. Espinosa-Marzal, Nanoheterogeneity of LiTFSI solutions transitions close to a surface and with concentration. *Nano Lett.* **21**, 2304–2309 (2021).

65. H. Weingärtner, The static dielectric constant of ionic liquids. *Z. Phys. Chem.* **220**, 1395–1405 (2006).

66. M. V. Fedorov, N. Georgi, A. A. Kornyshev, Double layer in ionic liquids: The nature of the camel shape of capacitance. *Electrochem. Commun.* **12**, 296–299 (2010).

67. A. A. Kornyshev, Double-layer in ionic liquids: Paradigm change? *J. Phys. Chem. B* **111**, 5545–5557 (2007).

68. Z. Gan, Y. Wang, Y. Lu, J. Qin, Y. Nie, H. He, Insight into the camel-to-bell transition of differential capacitance in ionic liquid-based supercapacitors. *ChemElectroChem* **9**, e202200274 (2022).

69. M. Heuberger, J. Vanicek, M. Zäch, The extended surface forces apparatus. II. Precision temperature control. *Rev. Sci. Instrum.* **72**, 3556–3560 (2001).

70. R. M. Espinosa-Marzal, T. Drobek, T. Balmer, M. P. Heuberger, Hydrated-ion ordering in electrical double layers. *Phys. Chem. Chem. Phys.* **14**, 6085–6093 (2012).

71. Z. Zachariah, R. M. Espinosa-Marzal, N. D. Spencer, M. P. Heuberger, Stepwise collapse of highly overlapping electrical double layers. *Phys. Chem. Chem. Phys.* **18**, 24417–24427 (2016).

72. J. E. Sader, J. W. M. Chon, P. Mulvaney, Calibration of rectangular atomic force microscope cantilevers. *Rev. Sci. Instrum.* **70**, 3967–3969 (1999).

73. J. M. Black, D. Walters, A. Labuda, G. Feng, P. C. Hillesheim, S. Dai, P. T. Cummings, S. V. Kalinin, R. Proksch, N. Balke, Bias-dependent molecular-level structure of electrical double layer in ionic liquid on graphite. *Nano Lett.* **13**, 5954–5960 (2013).

74. A. P. Thompson, H. M. Aktulga, R. Berger, D. S. Bolintineanu, W. M. Brown, P. S. Crozier, P. J. In't Veld, A. Kohlmeyer, S. G. Moore, T. D. Nguyen, LAMMPS—A flexible simulation tool for particle-based materials modeling at the atomic, meso, and continuum scales. *Comput. Phys. Commun.* **271**, 108171 (2022).

75. J. N. Canongia Lopes, J. Deschamps, A. A. Pádua, Modeling ionic liquids using a systematic all-atom force field. *J. Phys. Chem. B* **108**, 2038–2047 (2004).

76. L. Martinez, R. Andrade, E. G. Birgin, J. M. Martinez, PACKMOL: A package for building initial configurations for molecular dynamics simulations. *J. Comput. Chem.* **30**, 2157–2164 (2009).

77. G. Brug, A. L. van den Eeden, M. Sluyters-Rehbach, J. H. Sluyters, The analysis of electrode impedances complicated by the presence of a constant phase element. *J. Electroanal. Chem. Interfacial Electrochem.* **176**, 275–295 (1984).

78. B. Hirschorn, M. E. Orazem, B. Tribollet, V. Vivier, I. Frateur, M. Musiani, Determination of effective capacitance and film thickness from constant-phase-element parameters. *Electrochim. Acta* **55**, 6218–6227 (2010).

79. J. Song, M. Z. Bazant, Electrochemical impedance imaging via the distribution of diffusion times. *Phys. Rev. Lett.* **120**, 116001 (2018).

80. C. P. Fredlake, J. M. Crosthwaite, D. G. Hert, S. N. Aki, J. F. Brennecke, Thermophysical properties of imidazolium-based ionic liquids. *J. Chem. Eng. Data* **49**, 954–964 (2004).
